# Disulfide-Containing Nitrosoarenes: Synthesis and Insights into Their Self-Polymerization on a Gold Surface

**DOI:** 10.1021/acs.langmuir.4c03274

**Published:** 2024-12-09

**Authors:** Laura Nuić, Ana Senkić, Željka Car, Ena Asić, Nataša Vujičić, Marko Kralj, Ivana Biljan

**Affiliations:** † Department of Chemistry, Faculty of Science, University of Zagreb, Horvatovac 102A, HR-10000 Zagreb, Croatia; ∥ Center for Advanced Laser Techniques, Institute of Physics, Bijenička cesta 46, HR-10000 Zagreb, Croatia

## Abstract

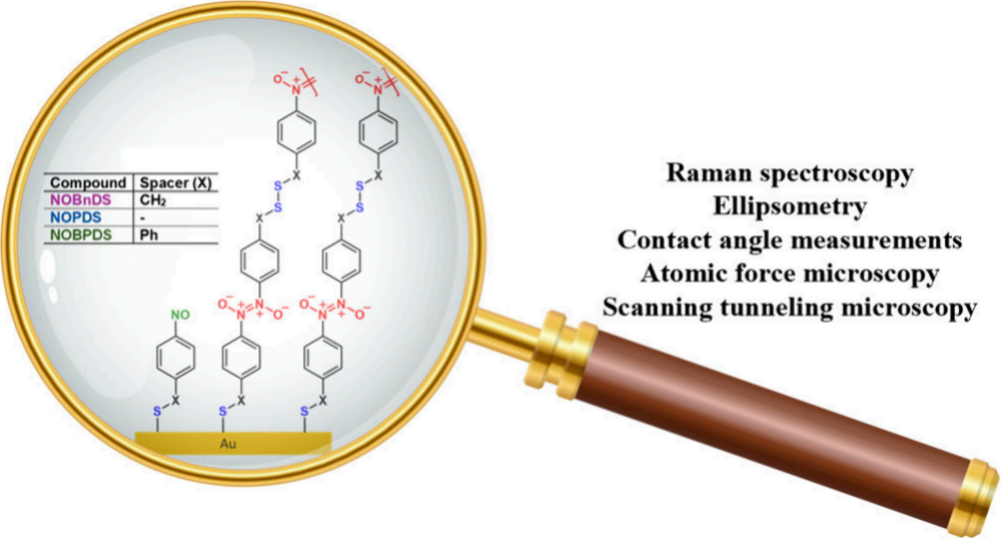

Disulfide-containing nitrosoarenes with [bis(4-nitrosobenzyl) disulfide, **NOBnDS** (**1**)] or without [4-nitrosophenyl disulfide, **NOPDS** (**2**), and 1,2-bis(4′-nitroso-[1,1′-biphenyl]-4-yl)disulfane, **NOBPDS** (**3**)] an alkyl spacer between the sulfur headgroup and the aromatic moiety (phenyl in **NOPDS** (**2**) or biphenyl in **NOBPDS** (**3**)) were synthesized and used as precursors to form azodioxy thiolate films on Au(111) substrates. Due to the incorporated disulfide functionalities, these specifically designed nitrosoarenes are enabled to self-polymerize through azodioxy bonds on a gold surface. Thin films of **NOBnDS** (**1**), **NOPDS** (**2**), and **NOBPDS** (**3**) were prepared at different adsorption times via the solution-phase self-assembly of molecules onto the Au(111) surface and characterized by Raman spectroscopy, ellipsometry, water contact angle measurements, atomic force microscopy (AFM), and scanning tunneling microscopy (STM). For comparison, films of structurally analogous disulfide-containing nitro derivatives on Au(111), which can form only monolayer films, thus serving as a reference, were also fabricated and characterized. Raman spectra of **NOBnDS** (**1**), **NOPDS** (**2**), and **NOBPDS** (**3**) films on the Au(111) substrates revealed the presence of a N=N stretching band of the *E*-azodioxy group. Ellipsometry showed that nitrosoarenes form thicker films compared with their nitro counterparts, indicating the formation of azodioxy oligomer films. AFM revealed an island-like morphology of the nitrosoarene films, in contrast to the rather homogeneous one observed for the monolayer films of nitro derivatives. STM images of nitrosoarene films indicated the formation of poorly organized surface structures in which oligomer chains are possibly interdigitated and substantially tilted relative to the surface normal, which agrees with the relatively low values of the ellipsometry-derived thicknesses. The obtained results emphasized the influence of the substituent electronic effects on the on-surface formation of azodioxides and could be used for the future design of azodioxy thiolate films on gold surfaces.

## Introduction

Aromatic C-nitroso compounds have diverse applications in coordination chemistry,^1−3^ synthetic organic chemistry^4−8^ as spin traps,^9−11^ and as reactive metabolites.^12,13^ This class of compounds is well known for its intriguing property to dimerize reversibly to *Z*- and *E*-azodioxides.^4,14^ Namely, in solution, nitroso monomer–azodioxide equilibrium exists, with monomers being the preferred form under ambient conditions.^4,14−20^ Azodioxides can be observed by lowering the solution temperature, with the *Z*-form usually predominating. In the solid state, aromatic C-nitroso compounds commonly occur as azodioxy dimers, mostly of the *E*-configuration. Especially interesting are derivatives with two or more nitrosoaryl groups due to their ability to form one-dimensional (1D), two-dimensional (2D), or three-dimensional (3D) azodioxy oligomers or polymers.^14,21−27^

For the effective application of aromatic C-nitroso compounds in fields such as synthesis, it is crucial to understand and carefully control the monomer–azodioxide equilibrium. Although the dimerization/polymerization property of nitrosoarenes in some cases presents obstacles for their usage, it can be exploited for the design of new functional materials such as molecular switches, organic semiconductors (OSCs), and crystalline porous organic networks.^4,14,21−23^ Previously, it was found that the azodioxy/nitroso system shows solid-state photochromic and thermochromic behavior.^28−35^ UV irradiation of aromatic azodioxides under cryogenic conditions in the solid phase yields nitroso monomers which undergo thermal redimerization to starting azodioxy compounds. Since photodissociation/dimerization is a repetitive process, this system acts as a potential molecular OFF–ON switch. Recent experimental and computational studies indicated that the azodioxy polymer of 1,4-dinitrosobenzene, poly(1,4-phenyleneazine-*N*,*N*-dioxide) (PNND), can be classified as a wide-band-gap OSC with potential applications in photodetectors and light-emitting diodes (LEDs).^22,23^ It is noteworthy that the adsorption of 1,4-dinitrosobenzene on the Au(111) surface results in significant narrowing of the band gap, allowing the possibility for the production of organic field-effect transistors (OFETs). Moreover, PNND was identified as the electrochemical oxidation product of Li_2_–BQDO (BQDO^2–^ stands for benzoquinone dioximate), which was investigated as an organic battery electrode material.^36^ Recently, reversible self-polymerization of monomers with tetrahedrally oriented nitroso groups was used for the construction of diamondoid azodioxy networks.^21^ In addition, a series of new aromatic trinitroso compounds were synthesized, and their polymerization to porous azodioxy networks with possible application for the adsorption of CO_2_, the main greenhouse gas, was evaluated.^37^

The adsorption of organic molecules on metal surfaces and their self-assembly into monolayer or multilayer films provides a way to design functional materials with a wide range of potential applications, e.g., in nanoelectronics, sensing, catalysis, lithography, and medicine.^38−50^ Surface properties of self-assembled monolayers (SAMs) and multilayers can be fine-tuned by incorporating different terminal functionalities, which can also act as anchoring sites for the attachment of other molecules or nanostructures. Due to their ability to dimerize and polymerize through azodioxy bonds, aromatic C-nitroso derivatives can be used for the systematic design of bilayer and multilayer films on surfaces. Indeed, our previous studies showed that the adsorption of nitrosoarenes containing a sulfur headgroup that strongly binds to gold surface results in the formation of nitroso monolayers and azodioxy bilayers on Au(111).^51−54^ Bilayers are produced by interactions of nitroso groups exposed at the monolayer interface and free nitroso molecules in solution. Scanning tunneling microscopy (STM) images revealed that mononitroso derivatives, e.g., 3-thiocyanatopropyl-4-nitrosobenzoate, form hexagonally ordered monolayer and bilayer surface structures on the Au(111) surface.^51^ The formation of bilayers was corroborated by atomic force microscopy (AFM) images and ellipsometry measurements.^51−54^ Although dinitrosoarenes, such as 3-thiocyanatopropyl-3,5-dinitrosobenzoate, also produce monolayers and bilayers as revealed by AFM, STM, and ellipsometry, these systems do not form ordered films on Au(111), indicating a significant impact of specific structural features of aromatic C-nitroso derivatives on their self-assembly on the gold surface.^53^ Studies investigating the influence of experimental parameters on the growth of monolayers and bilayers of nitrosoarenes on the Au(111) surface suggested the formation of more ordered films and a greater tendency toward bilayers with longer adsorption times and higher solution concentration.^54^ Recently, we studied the possible polymerization of a series of aromatic dinitroso derivatives initiated by a nitroso-terminated monolayer on the Au(111) surface at various adsorption times.^55^ The ellipsometry and AFM data suggested that the produced films are composed of only a few monomeric subunits or that poorly organized surface structures are formed with possibly tilted and intertwined chains. Nano-FTIR spectroscopy enabled nanoscale chemical identification of the films. Characteristic bands such as the stretching vibration of the *E*-azodioxy group were observed in nano-FTIR spectra recorded over isolated islands in topographic images.

**Figure 1:**
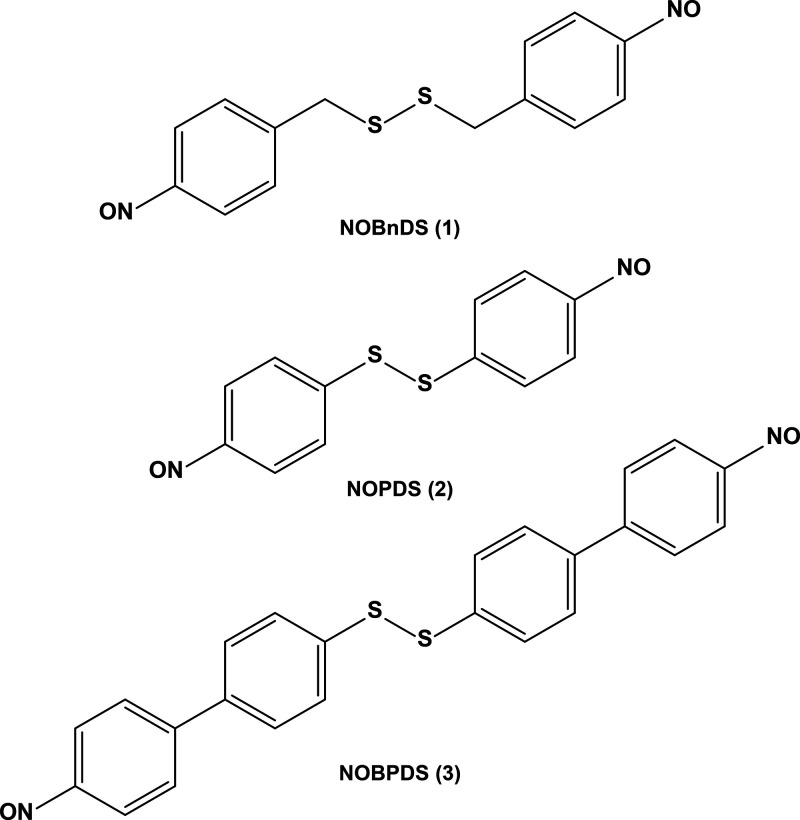
Molecular structures of new disulfide-containing nitrosoarenes: bis(4-nitrosobenzyl) disulfide, **NOBnDS** (**1**); bis(4-nitrosophenyl) disulfide, **NOPDS** (**2**); and 1,2-bis(4′-nitroso-[1,1′-biphenyl]-4-yl)disulfane, **NOBPDS** (**3**).

In the present study, we synthesized three new aromatic C-nitroso compounds **NOBnDS** (**1**), **NOPDS** (**2**), and **NOBPDS** (**3**) incorporating disulfide headgroups for adsorption on gold (Figure 1) and prepared their thin films via the solution-phase self-assembly of molecules onto the Au(111) surface.

**Figure 2:**
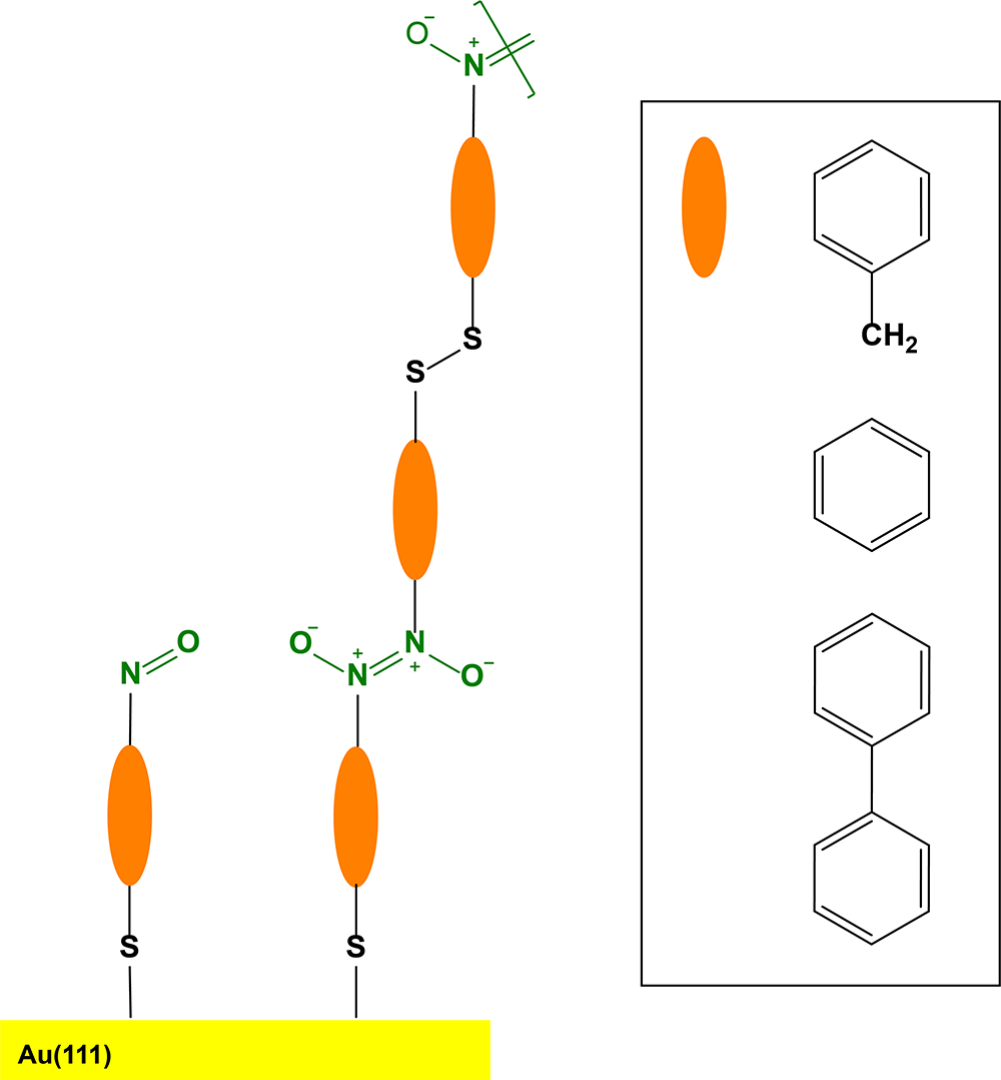
Possible self-polymerization of disulfide-containing nitrosoarenes **NOBnDS** (**1**), **NOPDS** (**2**), and **NOBPDS** (**3**) on the Au(111) surface and the formation of azodioxy films. The inset represents different molecular backbones in **NOBnDS** (**1**) (benzyl), **NOPDS** (**2**) (phenyl), and **NOBPDS** (**3**) (biphenyl) depicted by an orange ellipsoid.

Compound **NOBnDS** (**1**) contains a methylene group between the phenyl ring and the disulfide functionality. In **NOPDS** (**2**) and **NOBPDS** (**3**), differing in molecular backbone (phenyl vs biphenyl, respectively), the disulfide headgroups are directly attached to the aromatic moieties. Disulfide-containing nitrosoarenes **NOBnDS** (**1**), **NOPDS** (**2**), and **NOBPDS** (**3**) were specifically designed to study their adsorption and possible polymerization on the Au(111) substrate and the interrelationship between molecular and surface structures. While in the previous study nitrosoaryl groups exposed at the monolayer interface were used as initiation sites for the formation of azodioxy linkages by interactions with aromatic dinitroso derivatives prone to polymerization,^55^ herein-used nitrosoarene systems are enabled to self-polymerize due to incorporated disulfide functionalities (Figure 2).

However, dimerization and polymerization of aromatic C-nitroso compounds, among other things, are highly dependent on the structural and electronic effects. The presence of strong electron-donor substituents in the *para*-position with respect to the nitroso group usually prohibits or diminishes the formation of azodioxides due to the contribution of the quinoid resonance structure.^4^ In contrast to the previously studied films of aromatic C-nitroso derivatives containing alkyl chains of different lengths between the phenyl ring and sulfur headgroup, in this article, for the first time, films of nitrosoarenes with disulfide group directly attached to the aromatic ring (**NOPDS** (**2**) and **NOBPDS** (**3**)) were investigated. Such aromatic thiolate films are especially attractive due to their electronic properties and a wide range of potential applications in areas such as molecular electronics,^56−62^ charge transfer,^63^ lithography,^64−70^ etc. To analyze the chemical composition of **NOBnDS** (**1**), **NOPDS** (**2**), and **NOBPDS** (**3**) films on the Au(111) surface, their Raman spectra were recorded and compared with the bulk Raman spectra of powder samples. The topography and morphology of the **NOBnDS** (**1**), **NOPDS** (**2**), and **NOBPDS** (**3**) films prepared at different adsorption times on the Au(111) surface were investigated by ellipsometry, AFM, and STM, while wettability was determined by water contact angle measurements. For comparison, films of structural analogues, disulfide-containing nitro derivatives **NO**_**2**_**BnDS** (**1a**), **NO**_**2**_**PDS** (**2b**), and **NO**_**2**_**BPDS** (**3f**) on the Au(111) surface, were also fabricated and characterized.

## Experimental Section

### General Information

All chemicals were used as received from the suppliers. The solvents were purified or dried according to the literature procedures. The course of the reactions was monitored by thin-layer chromatography (TLC) (Merck silica gel 60-F_254_-coated plates). Column chromatography was performed with silica gel 60 (Fluka, 0.063–0.200 mm). Microwave-assisted synthesis was performed on a Discover SP microwave reactor equipped with an autosampler, IR temperature sensor, and integrated camera (CEM Corporation). The synthesized compounds were identified by ^1^H, ^13^C, and 2D ^1^H–^13^C HMBC nuclear magnetic resonance (NMR) spectroscopy, infrared (IR) spectroscopy, and mass spectrometry (MS). ^1^H and ^13^C NMR spectra were recorded on a Bruker Avance III HD 400 MHz/54 mm Ascend spectrometer in deuterated chloroform (CDCl_3_) or deuterated dimethyl sulfoxide-*d*_6_ (DMSO-*d*_6_) at 298 K. ^1^H–^13^C HMBC spectra were recorded on a Bruker Avance III HD 600 MHz/54 mm Ascend spectrometer in DMSO-*d*_6_ at 298 K. The chemical shifts (δ) are reported as ppm relative to tetramethylsilane (TMS). The multiplet signal is reported as s–singlet, d–doublet, t–triplet, or m–multiplet. IR spectra were recorded on a PerkinElmer UATR Two spectrometer in the spectral range between 4000 and 400 cm^–1^ at a resolution of 4 cm^–1^, averaging 10 scans per spectrum. Mass spectrometric analysis was conducted in ESI mode on a high-resolution Agilent 6550 accurate-mass-quadrupole time-of-flight (Q-TOF) spectrometer.

### Synthesis

**Figure 3:**
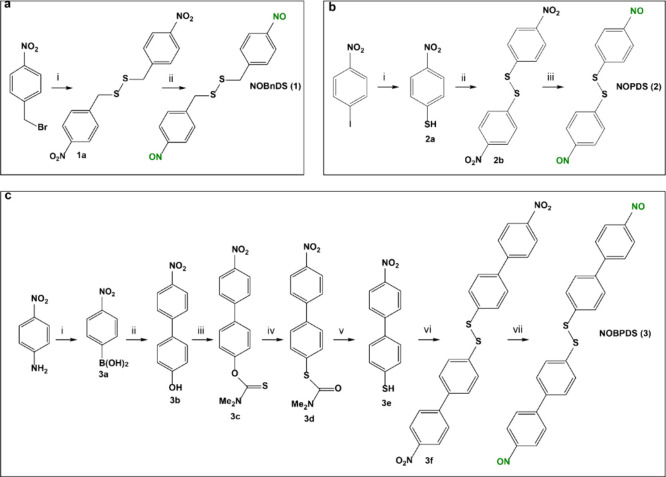
Synthesis routes to (a) **NOBnDS** (**1**), (b) **NOPDS** (**2**), and (c) **NOBPDS** (**3**). Reactions in a: (i) DMSO:H_2_O = 10:1, Na_2_S_2_O_3_·5H_2_O, 65 °C, 45 min, 56%; (ii) 1) DME, NH_4_Cl, Zn, rt, 1 h; 2) FeCl_3_·6H_2_O, −5 °C, 32%. Reactions in b: (i) 1) DMF, S, K_2_CO_3_, 75 °C, 4 h, Ar; 2) Ph_3_P, dioxane:H_2_O:2 M HCl = 2:2:1, 45 °C, 24 h, Ar, 54%; (ii) EtOAc, NaI, 30% H_2_O_2_, rt, 45 min, 87%; (iii) 1) acetone, NH_4_Cl, Zn, rt, 30 min; 2) FeCl_3_·6H_2_O, −5 °C, 18%. Reactions in c: (i) 1) MeOH, HCl, H_2_O, NaNO_2_, 0 °C, 75 min; 2) B_2_(OH)_4_, rt, 90 min, 72%; (ii) toluene, Pd(PPh_3_)_4_, 2 M Na_2_CO_3_, 80 °C, 3 h, N_2_, 28%; (iii) 1) DMF, 90% NaH, 0 °C, N_2_, 12 h; 2) Me_2_NCSCl, 60 °C, 12 h, N_2_, 46%; (iv) MW, DMA, 250 °C, 35 min, 59%; (v) MeOH, KOH, 80 °C, 6 h, N_2_, 89%; (vi) EtOAc, 1 mol % NaI, 30% H_2_O_2_, rt, 35 min, 96%; (vii) 1) acetone, NH_4_Cl, Zn, rt, 1 h; 2) Ag_2_CO_3_/Celite, −5 °C, 5 min, 8%.

Compounds **1a**, **2a**, **2b**, **3a**, **3b**, **3c**, **3d**, **3e**, and **3f** were synthesized by slightly modified procedures from those reported.^71−76^ Nitroso derivatives **NOBnDS** (**1**), **NOPDS** (**2**), and **NOBPDS** (**3**) were prepared from corresponding nitro derivatives **NO**_**2**_**BnDS** (**1a**), **NO**_**2**_**PDS** (**2b**), and **NO**_**2**_**BPDS** (**3f**) by standard procedures based on Zn reduction followed by oxidation with FeCl_3_ (compounds **1** and **2**) or Ag_2_CO_3_/Celite (compound **3**). The synthesis routes for compounds **NOBnDS** (**1**), **NOPDS** (**2**), and **NOBPDS** (**3**) are shown in Figure 3.

### Bis(4-nitrosobenzyl) Disulfide, **NOBnDS** (**1**)

The synthesized compound **1a** (0.625 mmol) was dissolved in acetone (5 mL). The solution of NH_4_Cl (4.12 mmol) in distilled H_2_O (3.5 mL) was added first, and then preactivated zinc powder (12 mmol) was added in small portions to the reaction mixture while stirring vigorously. After all of the zinc was added, the mixture was stirred for 1 h at room temperature and then filtered. An ice-cold solution of FeCl_3_·6H_2_O (4.68 mmol) in distilled H_2_O (11 mL) and ethanol (5 mL) was added to the cold filtrate (−5 °C). The mixture was extracted with EtOAc (2×). The organic layer was dried over anhydrous Na_2_SO_4_. After the evaporation of the solvent, the obtained solid was purified by column chromatography, eluting with petroleum ether:ethyl acetate = 4:1 to afford a pure product as a green oil which solidified upon refrigeration as a yellowish solid with a yield of 32%. ^1^H (400 MHz, CDCl_3_) δ/ppm: 7.87 (d, 2H, *J* = 8.3 Hz); 7.45 (d, 2H, *J* = 8.3 Hz); 3.68 (s, 2H). ^13^C (100 MHz, CDCl_3_) δ/ppm: 165.2 (C–NO); 145.8 (C); 130.5 (CH–Ph); 121.6 (CH–Ph); 43.1 (CH_2_). IR (ATR) ν̃/cm^–1^: 3052 (=C–H); 1598 (C=C); 1506 (NO); 1261 (*E*–ON=NO); 1112 (C–N); 511 (S–S). MS (ESI) *m*/*z*: Calculated [M] for C_14_H_12_N_2_O_2_S_2_ 304.0340 and found [M–H]^−^ = 303.0272.

### 4-Nitrosophenyl Disulfide, **NOPDS** (**2**)

The synthesized compound **2b** (0.649 mmol) was dissolved in acetone (5 mL). The solution of NH_4_Cl (4.28 mmol) in distilled H_2_O (3.5 mL) was added first, and then the preactivated zinc powder (9.47 mmol) was added in small portions to the reaction mixture while stirring vigorously. After all of the zinc was added, the mixture was stirred for 30 min at room temperature and then filtered. An ice-cold solution of FeCl_3_·6H_2_O (4.86 mmol) in distilled H_2_O (11 mL) and ethanol (5 mL) was added to the cold filtrate (−5 °C). The mixture was extracted with EtOAc (2×). The organic layer was dried over anhydrous Na_2_SO_4_. After the evaporation of the solvent, the obtained solid was purified by column chromatography, eluting with DCM to afford a pure product as a green solid with a yield of 18%. ^1^H (400 MHz, CDCl_3_) δ/ppm: 7.86 (d, 4H, *J* = 8.5 Hz); 7.70 (d, 4H, *J* = 8.5 Hz). ^13^C (100 MHz, CDCl_3_) δ/ppm: 164.7 (C–NO); 145.3 (C); 126.5 (CH–Ph); 122.1 (CH–Ph). IR (ATR) ν̃/cm^–1^: 3093 (=C–H); 1575 (C=C); 1484 (NO); 1261 (*E*–ON=NO); 1113 (C–N); 512 (S–S). MS (ESI) *m*/*z*: Calculated [M] for C_12_H_8_N_2_O_2_S_2_ 276.0027 and found [M–H]^−^ 274.9952.

### 1,2-Bis(4′-nitroso-[1,1′-biphenyl]-4-yl)disulfane, **NOBPDS** (**3**)

The synthesized compound **3f** (0.738 mmol) was dissolved in acetone (8 mL). The solution of NH_4_Cl (2.5 mmol) in distilled H_2_O (2 mL) was added first, and then the preactivated zinc powder (5.9 mmol) was added in small portions to the reaction mixture while stirring vigorously. After all of the zinc was added, the mixture was stirred for 1 h at room temperature and then filtered. The filtrate was cooled to −5 °C, and Ag_2_CO_3_ on Celite (5.9 mmol) was added. The reaction mixture was stirred for 5 min at room temperature. The reaction mixture was extracted with DCM (2×) and dried over anhydrous Na_2_SO_4_. After the evaporation of the solvent, the obtained solid was purified by column chromatography, eluting with chloroform to afford a pure product as a green oil, which solidified upon refrigeration as a yellowish solid with a yield of 8%. ^1^H (400 MHz, CDCl_3_) δ/ppm: 7.97 (d, 4H, *J* = 8.5 Hz); 7.80 (d, 4H, *J* = 8.5 Hz); 7.63–7.65 (m, 8H). ^13^C (100 MHz, CDCl_3_) δ/ppm: 165.1 (C–NO); 147.2 (C–S); 138.5 (C); 138.3 (C); 128.5 (CH–Ph); 128.1 (CH–Ph); 127.9 (CH–Ph); 124.6 (CH–Ph). IR (ATR) ν̃/cm^–1^: 2923 (=C–H); 1596 (C=C); 1480 (NO); 1259 (*E*–ON=NO); 1081 (C–N); 512 (S–S). MS (ESI) *m*/*z*: Calculated [M] for C_24_H_16_N_2_O_2_S_2_ 428.0653 and found [M + H]^+^ 429.0731.

### Preparation of Films on the Au(111) Surface

All glassware was cleaned using piranha solution (3:1 mixture of sulfuric acid and 30% hydrogen peroxide heated to 90 °C). Previously flame-annealed Au(111)/mica substrates (Phasis) were immersed in a 1 mM solution of **NOBnDS** (**1**), **NOPDS** (**2**), or **NOBPDS** (**3**) in a 1:1 mixture of chloroform and ethanol for 24 or 48 h at room temperature. The as-prepared films of **NOBnDS** (**1**) and **NOPDS** (**2**) were also postannealed in solution at 328 K for 2 h. Films of nitroarene derivatives **NO**_**2**_**BnDS** (**1a**), **NO**_**2**_**PDS** (**2b**), and **NO**_**2**_**BPDS** (**3f**) were prepared by immersing previously flame-annealed Au(111)/mica substrates (Phasis) in a 1 mM solution of the corresponding compound for 24 h. After being removed from the solution, the substrate was thoroughly rinsed with a copious amount of solvent and dried under argon.

### Raman Measurements

All Raman spectra were taken with a commercial Renishaw in-via confocal microscope. It is equipped with a 532 nm (2.33 eV) continuous wave laser source. All measurements were taken with a 50× objective (NA = 0.50) and a grating with a 2400 mm^–1^ constant. For samples in their powder form, the acquisition time was 5 × 1 s and the laser power on the sample was 83 μW. In the case of **NOBnDS** (**1**) on the Au substrate, the acquisition time was 10 × 2 s under a laser power of 1 mW. For **NOPDS** (**2**) on the Au substrate, due to its small concentration, the laser power was increased to 3.45 mW while the acquisition time was 20 × 1 s. Finally, for the **NOBPDS (3)** on the Au substrate, the acquisition time was 20 × 5 s under a laser power of 1 mW.

### Ellipsometry Measurements

All ellipsometry measurements were performed using an L116B ellipsometer (Gaertner Scientific) with a rotating analyzer and a 632.8 nm HeNe laser with a light incident angle of 70°. All measurements were conducted under ambient conditions and humidity of 30–50%. The optical constants for gold (N_s_ and K_s_ refractive index values) were measured after the flame annealing procedure at a minimum of ten different areas on each sample. The value of 1.55 was used for the index of refraction.^77^ Ellipsometry measurements were carried out on a minimum of 20 different areas on each sample (sample size 1 × 1 cm). Measurements of thickness were carried out with the GEMP program.

### Contact Angle Measurements

Static contact angles of 4 to 5 μL drops of 2 mS/cm H_2_O were measured on a minimum of five different spots on the surface with an easy drop Theta Lite Optical Tensiometer instrument.

### AFM Measurements

AFM measurements were conducted on a MultiMode 8 (Bruker) using tapping mode with silicon tips (Bruker, NCHV-A, nom. spring constant 40 N/m, nom. freq 320 kHz). All measurements were performed under ambient conditions and humidity of 30–50%. Processing and analysis of data were carried out with NanoScope Analysis 2.0 (Bruker) software.

### STM Measurements

STM measurements were performed using MultiMode 8 (Bruker) and commercially available Pt/Ir tips (Bruker, 0.25 mm diameter). All STM images were obtained in the constant-current mode using tunneling currents ranging from 10 pA to 1 nA and bias voltage in the range of 0.5 to 1 V. All measurements were conducted under ambient conditions and humidity of 30–50%. Processing and analysis of data were carried out by using the NanoScope Analysis 2.0 (Bruker) software.

## Results and Discussion

### Synthesis and Structural Characterization of **NOBnDS** (**1**), **NOPDS** (**2**), and **NOBPDS** (**3**)

The target nitroso compounds **NOBnDS** (**1**), **NOPDS** (**2**), and **NOBPDS** (**3**), were synthesized by the reduction of the corresponding nitro derivatives **NO**_**2**_**BnDS** (**1a**), **NO**_**2**_**PDS** (**2b**), and **NO**_**2**_**BPDS** (**3f**) to *N*-arylhydroxylamines, followed by their in situ oxidation (Figure 3). For the synthesis of **NO**_**2**_**BnDS** (**1a**), 4-nitrobenzyl bromide was reacted with sodium thiosulfate, resulting in the formation of the corresponding Bunte salt (Figure 3a). Self-catalyzed hydrolysis of the Bunte salt yielded 4-nitrobenzyl thiol, which underwent oxidative coupling with dimethyl sulfoxide to afford **NO**_**2**_**BnDS** (**1a**). For the synthesis of **NO**_**2**_**PDS** (**2b**), 1-iodo-4-nitrobenzene was reacted with sulfur, followed by the reduction with triphenylphosphine to thiol derivative **2a**, which was then oxidized by hydrogen peroxide in the presence of a catalytic amount of sodium iodide to **NO**_**2**_**PDS** (**2b**) (Figure 3b). The synthesis of **NO**_**2**_**BPDS** (**3f**) started with the preparation of compound **3a** by conducting the diazotization of 4-nitroaniline, followed by the borylation reaction without isolating the diazonium salt intermediate (Figure 3c). The Suzuki-Miyaura coupling reaction between **3a** and commercially available 4-bromophenol gave compound **3b**, which was then treated with *N*,*N*-dimethylthiocarbamoyl chloride in the presence of sodium hydride, resulting in the formation of compound **3c**. In the next step, **3c** underwent the microwave-assisted Newman-Kwart rearrangement to form the corresponding *S*-thiocarbamate **3d**. It is noteworthy that, to the best of our knowledge, this type of rearrangement was successfully optimized and carried out under microwave conditions for the first time on nitro-substituted biphenyls and could be used for the synthesis of similar derivatives. The reaction was optimized regarding the reaction time, solvent, temperature, and amount of reactant used (Table S1). The hydrolysis of compound **3d** yielded thiol-terminated **3e**, which was then oxidized to disulfide-containing **NO**_**2**_**BPDS** (**3f**).

**Figure 4:**
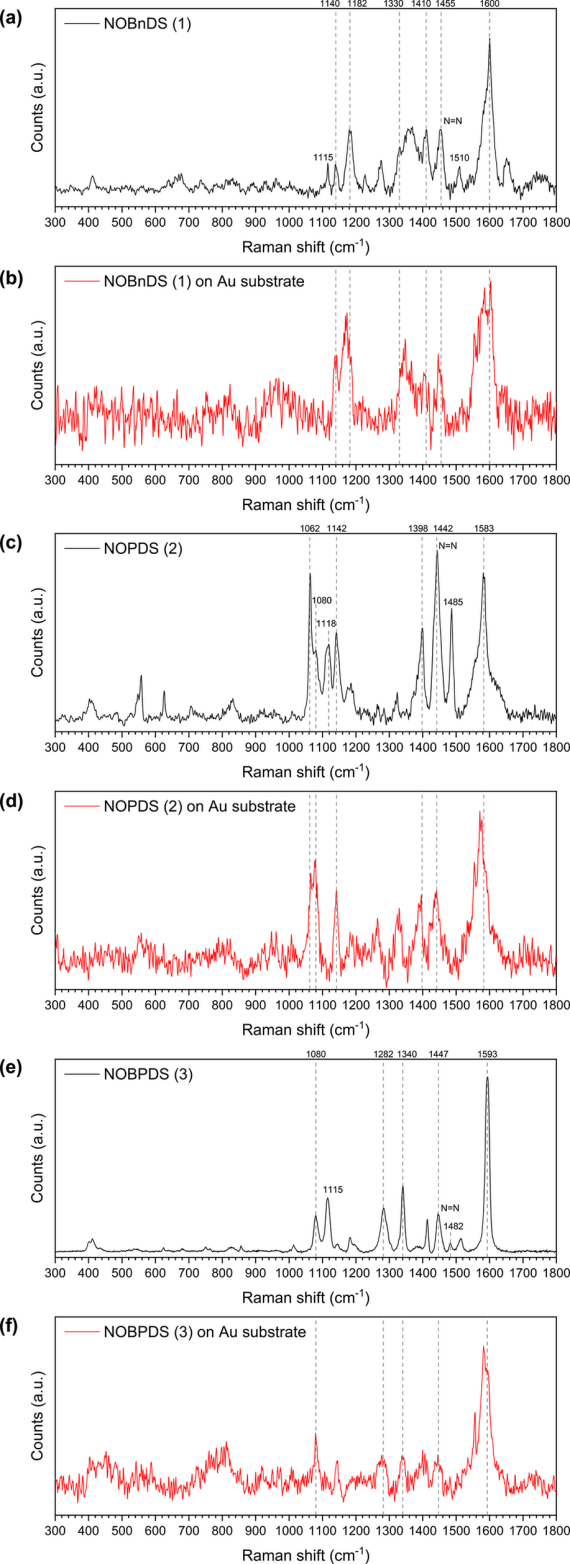
Raman spectra of (a) **NOBnDS** (**1**), (c) **NOPDS** (**2**), and (e) **NOBPDS** (**3**) in powder form and films prepared by immersion of the Au(111) substrate in a 1 mM solution of (b) **NOBnDS** (**1**), (d) **NOPDS** (**2**), and (f) **NOBPDS** (**3**) for 24 h. The dashed vertical lines mark the matching frequencies in the bulk Raman spectra of powder samples and the spectra of films.

Due to the possible intermolecular reactions of two nitroso groups of **NOBnDS** (**1**), **NOPDS** (**2**), and **NOBPDS** (**3**), these compounds can appear as *Z*- or *E*-azodioxy dimers, oligomers, or polymers. An inspection of the IR spectra of compounds **NOBnDS** (**1**), **NOPDS** (**2**), and **NOBPDS** (**3**) revealed the presence of bands at 1506 and 1112 cm^–1^ (for **NOBnDS** (**1**)), at 1484 and 1113 cm^–1^ (for **NOPDS** (**2**)), and at 1480 and 1081 cm^–1^ (for **NOBPDS** (**3**)), attributed to nitroso monomer N=O and C–N stretching vibrations, respectively (Figures S2, S5, and S12). In the spectra of all three compounds, a band at 1261 cm^–1^ (for **NOBnDS** (**1**) and **NOPDS** (**2**)) and at 1259 cm^–1^ (for **NOBPDS** (**3**)) was detected, which can be assigned to the asymmetric stretching vibration of the *E*-azodioxy group. The IR bands characteristic of the *Z*-azodioxides were not observed in the spectra of **NOBnDS** (**1**), **NOPDS** (**2**), and **NOBPDS** (**3**). These data suggested that **NOBnDS** (**1**), **NOPDS** (**2**), and **NOBPDS** (**3**) are isolated as *E*-azodioxy oligomers in the solid state with a visible number of nitroso end-groups, with **NOPDS** (**2**) having a higher nitroso monomer/*E*-azodioxy oligomer signal ratio compared to **NOBnDS** (**1**) and **NOBPDS** (**3**). This can be explained by the electron-donating sulfur atom in the *para*-position relative to the nitroso group of **NOPDS** (**2**) stabilizing the quinoid resonance structure.^4^ IR spectroscopy data of **NOBnDS** (**1**), **NOPDS** (**2**), and **NOBPDS** (**3**) were supplemented by complementary Raman spectroscopy. The Raman spectra of **NOBnDS** (**1**), **NOPDS** (**2**), and **NOBPDS** (**3**) contain bands at 1455, 1442, and 1447 cm^–1^, respectively, attributed to the N=N stretching vibration of *E*-azodioxides, which is Raman- but not IR-active (Figure 4a, c, and e).^78,79^ In addition, the Raman spectra of all three compounds revealed bands at around 1500 and 1100 cm^–1^, which probably correspond to nitroso monomer N=O and C–N stretching vibrations. These bands are situated at 1510 and 1115 cm^–1^ for **NOBnDS** (**1**), at 1485 and 1118 cm^–1^ for **NOPDS** (**2**), and at 1482 and 1115 cm^–1^ for **NOBPDS** (**3**). The bands attributed to nitroso monomer vibrations are more intense in the Raman spectra of **NOPDS** (**2**) compared to the spectra of **NOBnDS** (**1**) and **NOBPDS** (**3**), which further supports the previous conclusion based on the IR spectroscopy data that **NOPDS** (**2**) possesses a higher proportion of monomers than the other two studied nitrosarenes. In solution under ambient conditions, **NOBnDS** (**1**), **NOPDS** (**2**), and **NOBPDS** (**3**) appear exclusively as nitroso monomers as indicated by an inspection of their solution-state ^1^H and ^13^C NMR spectra (Figures S15, S16, S21, S22, S36, and S37).

### Raman Spectra of **NOBnDS** (**1**), **NOPDS** (**2**), and **NOBPDS** (**3**) Films on the Au(111) Surface

To analyze the chemical composition of the **NOBnDS** (**1**), **NOPDS** (**2**), and **NOBPDS** (**3**) films formed after 24 h on the Au(111) substrates, their Raman spectra were recorded and compared with the bulk Raman spectra of powder samples (Figure 4). Direct comparison of the Raman spectra of the films on the Au substrates and the bare Au substrates can be found in the Supporting Information (Figure S38). In all three cases, the intensities of the Raman spectra were heavily reduced after the adsorption of **NOBnDS** (**1**), **NOPDS** (**2**), and **NOBPDS** (**3**) molecules on the Au(111) substrate. Since the samples’ concentration on the Au substrate is very small, this behavior is expected. Nevertheless, a comparison of the bulk Raman spectra of **NOBnDS** (**1**), **NOPDS** (**2**), and **NOBPDS** (**3**) and their films on the Au substrates revealed that the frequencies of several typical vibrational modes coincide (the matching frequencies are marked with dashed vertical lines in Figure 4), which confirmed the presence of molecules on the Au substrate. The most intense bands in the Raman spectra of **NOBnDS** (**1**), **NOPDS** (**2**), and **NOBPDS** (**3**) films are situated at 1600, 1583, and 1593 cm^–1^, respectively, and can be assigned to the aromatic vibration.^80,81^ In addition, the Raman spectra of **NOPDS** (**2**) and **NOBPDS** (**3**) films showed bands at 1080 cm^–1^, which probably correspond to the in-plane ring breathing mode coupled with the C–S vibration.^80,81^ The prominent feature in the Raman spectra of **NOBnDS** (**1**), **NOPDS** (**2**), and **NOBPDS** (**3**) films was the appearance of the N=N stretching vibration band at 1455, 1442, and 1447 cm^–1^, respectively, characteristic of *E*-azodioxides.^78,79^ The bands attributed to nitroso monomer N=O and C–N stretching vibrations, which are present in the bulk Raman spectra of powder samples, were not detected in the spectra of their films on the Au substrates. These data indicated that the self-assembly of **NOBnDS** (**1**), **NOPDS** (**2**), and **NOBPDS** (**3**) molecules from the solutions in which they are present as nitroso monomers resulted in the formation of *E*-azodioxides on the Au(111) surface. To record the Raman spectra of **NOPDS** (**2**) films on the Au substrate, the laser power had to be significantly increased compared to those of **NOBnDS** (**1**) and **NOBPDS** (**3**), which could suggest the formation of thinner films in the former case.

### Ellipsometry and Contact Angles

**Figure 5:**
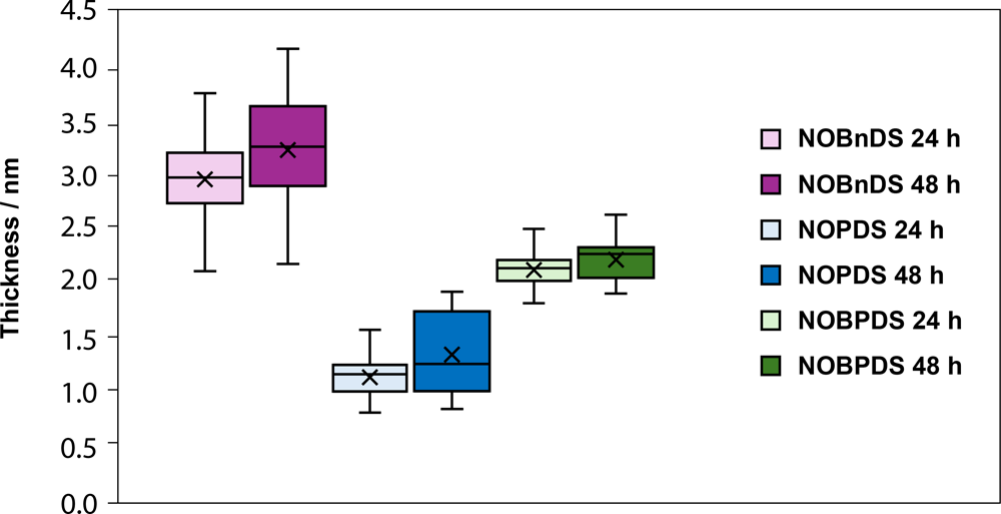
Ellipsometry thicknesses of films produced by immersion of a clean Au(111) substrate in a 1 mM solution of **NOBnDS** (**1**), **NOPDS** (**2**), and **NOBPDS** (**3**) for 24 or 48 h. Data are presented as box plots with the boxes displaying 25th to 75th percentiles (the distance between the upper and lower lines of the box is the interquartile range (IQR)), the horizontal lines within the boxes are the median values, the lines outside the boxes represent the range within 1.5 IQR, and mean values are depicted by “x” markers.

Ellipsometry measurements were conducted to determine the film thicknesses of **NOBnDS** (**1**), **NOPDS** (**2**), and **NOBPDS** (**3**) on Au(111). Nitroso groups exposed at the monolayer interface could interact with nitroso molecules in solution via azodioxy bonds, thus creating dimeric, oligomeric, or polymeric surface structures. For comparison, we also prepared and characterized films of structurally analogous disulfide-containing nitro derivatives **NO**_**2**_**BnDS** (**1a**), **NO**_**2**_**PDS** (**2b**), and **NO**_**2**_**BPDS** (**3f**) on the Au(111) surface, which can form only monolayers (SAMs) and thus served as a reference. The thicknesses of the nitro-terminated SAMs of **NO**_**2**_**BnDS** (**1a**), **NO**_**2**_**PDS** (**2b**), and **NO**_**2**_**BPDS** (**3f**) formed after 24 h were measured to be ∼1.07, ∼ 0.92, and ∼1.30 nm, respectively (Figure S39). The obtained values for **NO**_**2**_**PDS** (**2b**) and **NO**_**2**_**BPDS** (**3f**) monolayers are in good agreement with the film thicknesses previously derived from the XPS data for the SAMs of a similar system of 4-nitrophenyl-1-thiol (PT-NO_2_) and 4′-nitrobiphenyl-4-thiol (BPT-NO_2_), which is ∼0.89 nm for PT-NO_2_ and ∼1.35 nm for BPT-NO_2_.^82^ Namely, the adsorption of organic thiols and disulfides on the gold surface results in SAMs of similar structures due to the cleavage of disulfide bond upon adsorption on the gold surface.^39^ Comparison with the estimated molecular lengths of **NO**_**2**_**PDS** (**2b**) and **NO**_**2**_**BPDS** (**3f**) of ∼0.68 and ∼1.14 nm, respectively, and a consideration of the S–Au bond length of 0.24–0.25 nm^82,83^ suggests that in the corresponding SAMs the molecules are in an upright orientation. The experimentally determined thickness of the **NO**_**2**_**BnDS** (**1a**) SAM (∼1.07 nm) is quite close to the expected value (∼1.06 nm), also indicating a perpendicular molecular orientation. This observation correlates well with the previous studies of benzylmercaptan and *p*-cyanobenzylmercaptan monolayers on Au(111) which showed that both molecules form well-ordered SAMs with molecules oriented almost perpendicular to the surface.^84−86^ Overall, ellipsometry data for the films of nitroarenes **NO**_**2**_**BnDS** (**1a**), **NO**_**2**_**PDS** (**2b**), and **NO**_**2**_**BPDS** (**3f**) indicated the presence of exclusively monolayers on the Au(111) surface, which allows their use as good reference systems for studying films of nitroso derivatives on Au(111). Figure 5 shows ellipsometry-derived thicknesses of **NOBnDS** (**1**), **NOPDS** (**2**), and **NOBPDS** (**3**) films formed by immersing a freshly annealed Au(111) substrate in a 1 mM solution of the corresponding nitroso compound for 24 and 48 h. The highest values were observed for films of **NOBnDS** (**1**), which contain a methylene unit between the phenyl ring and the disulfide group. The film thicknesses of **NOBnDS** (**1**) after 24 h are distributed in the range from 2.07 to 3.72 nm with a median value of 2.93 nm indicating the formation of significantly thicker films when compared to the nitro analogue **NO**_**2**_**BnDS** (**1a**) (∼1.07 nm). These results suggested the formation of azodioxy oligomer films of **NOBnDS** (**1**) on the Au(111) surface. The thickness values for the aromatic **NOPDS** (**2**) and **NOBPDS** (**3**) films are spread from 0.77 to 1.53 nm (median value is 1.09 nm) and from 1.78 to 2.47 nm (median value is 2.09 nm), respectively, for an adsorption time of 24 h. The obtained values for **NOPDS** (**2**) and **NOBPDS** (**3**) films are higher compared to the film thicknesses of the corresponding nitro analogues **NO**_**2**_**PDS** (**2b**) (∼0.92 nm) and **NO**_**2**_**BPDS** (**3f**) (∼1.30 nm). However, the difference in thickness between **NOPDS** (**2**) and **NO**_**2**_**PDS** (**2b**) films is less pronounced compared to those of the **NOBnDS** (**1**)-**NO**_**2**_**BnDS** (**1a**) and **NOBPDS** (**3**)-**NO**_**2**_**BPDS** (**3f**) pairs. This suggested a lower tendency for on-surface oligomerization of **NOPDS** (**2**) with disulfide groups directly attached to the aromatic ring and in the *para*-position relative to the nitroso group in comparison to compound **NOBnDS** (**1**) with a methylene unit inserted between the phenyl ring and disulfide headgroup. For all three studied nitroso compounds, the prolongation of the adsorption time to 48 h did not result in a significant increase in the median film thicknesses (Figure 5).

Table 1 shows water contact angles of **NOBnDS** (**1**), **NOPDS** (**2**), and **NOBPDS** (**3**) films formed on the Au(111) surface after 24 and 48 h. Evidently, a longer adsorption time does not have an observable effect on the wettability of the films. The contact angles of **NOPDS** (**2**) films are somewhat smaller than those of **NOBnDS** (**1**) and **NOBPDS** (**3**) films, which could be attributed to a lower tendency for the oligomerization of **NOPDS** (**2**) as also suggested by the ellipsometry data. When compared to alkylthiolate films on Au(111),^87−90^ the contact angles of **NOBnDS** (**1**), **NOPDS** (**2**), and **NOBPDS** (**3**) films are smaller, which is in accordance with literature data for similar systems containing aromatic moieties.^91−94^ Monolayers of nitroarenes **NO**_**2**_**BnDS** (**1a**), **NO**_**2**_**PDS** (**2b**), and **NO**_**2**_**BPDS** (**3f**) displayed lower contact angle values (Table S2) compared to films of the corresponding nitroso derivatives on Au(111), indicating their more hydrophilic nature due to exposed polar nitro groups. The herein-determined water contact angle for the **NO**_**2**_**BPDS** (**3f**) film is in good agreement with the literature value of 64°.^91^ The obtained results agree with the ellipsometry and Raman spectroscopy data, indicating the formation of azodioxy oligomer films of studied nitroso derivatives on Au(111).

**Table 1:** Static Water Contact Angles of **NOBnDS** (**1**), **NOPDS** (**2**), and **NOBPDS** (**3**) Films Formed on the Au(111) Surface after 24 and 48 h

compound	**NOBnDS** (**1**)		**NOBnDS** (**2**)		**NOBnDS** (**3**)	
adsorption time	24 h	48 h	24 h	48 h	24 h	48 h
average contact angle	71 ± 3°	70 ± 4°	65 ± 3°	65 ± 3°	73 ± 3°	74 ± 3°

### AFM

**Figure 6:**
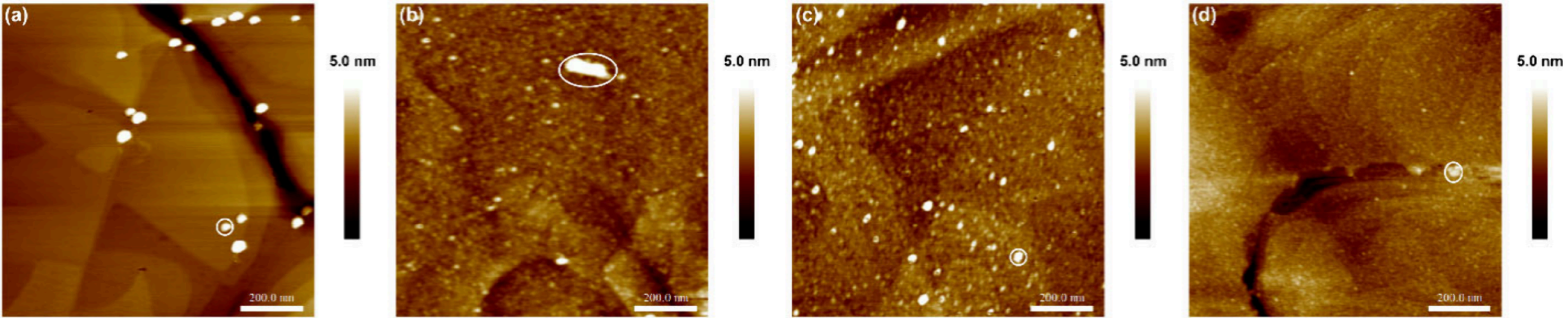
1 μm × 1 μm AFM images of (a) a bare Au(111) surface and films prepared by immersion of the Au(111) substrate in a 1 mM solution of compound (b) **NOBnDS** (**1**), (c) **NOPDS** (**2**), or (d) **NOBPDS** (**3**) for 24 h. Bright clusters appearing on the surface of a clean Au(111) substrate and after the adsorption of molecules (marked with white circles) are attributed to environmental contaminants under ambient conditions and surface preparation treatment.

The morphology and topography of **NOBnDS** (**1**), **NOPDS** (**2**), and **NOBPDS** (**3**) films on the Au(111) surface was investigated by AFM. Figure 6 shows the AFM images of the bare gold surface (Figure 6a) and gold surface modified by **NOBnDS** (**1**), **NOPDS** (**2**), and **NOBPDS** (**3**) (Figure 6b–6d). Evidently, the immersion of a clean Au(111) substrate in a 1 mM solution of **NOBnDS** (**1**), **NOPDS** (**2**), and **NOBPDS** (**3**) induced significant changes in the surface morphology. When compared to monolayers of nitro derivatives **NO**_**2**_**BnDS** (**1a**), **NO**_**2**_**PDS** (**2b**), and **NO**_**2**_**BPDS** (**3f**) on the Au(111) surface, the differences in surface morphology are visible (Figure S40). Namely, while **NO**_**2**_**BnDS** (**1a**), **NO**_**2**_**PDS** (**2b**), and **NO**_**2**_**BPDS** (**3f**) form rather homogeneous films on Au(111) attributed to SAMs, films of nitroso derivatives **NOBnDS** (**1**), **NOPDS** (**2**), and **NOBPDS** (**3**) appear to be more heterogeneous, displaying island-like morphology. According to the literature, the highest protruding features, i.e., the bright cluster which can be observed on the gold surface before and after the adsorption of nitroso and nitro molecules (marked with white circles in Figures 6, S40, and S41), are attributed to environmental contaminants under ambient conditions and surface preparation treatment.^53,54,95^

The appearance of small islands in the films of nitroso derivatives **NOBnDS** (**1**), **NOPDS** (**2**), and **NOBPDS** (**3**), in accordance with previous studies,^51−55^ pointed to the formation of azodioxides on the gold surface and corroborates well with the results of Raman, ellipsometry, and contact angle measurements. The immersion of Au(111) substrate in a 1 mM solution of **NOBnDS** (**1**), **NOPDS** (**2**), and **NOBPDS** (**3**) for a longer adsorption time of 48 h did not induce significant changes in the morphology of the formed films, which is again characterized by the presence of a high number of small islands (Figure S41) and is in good agreement with ellipsometry and contact angle data.

### STM

**Figure 7:**
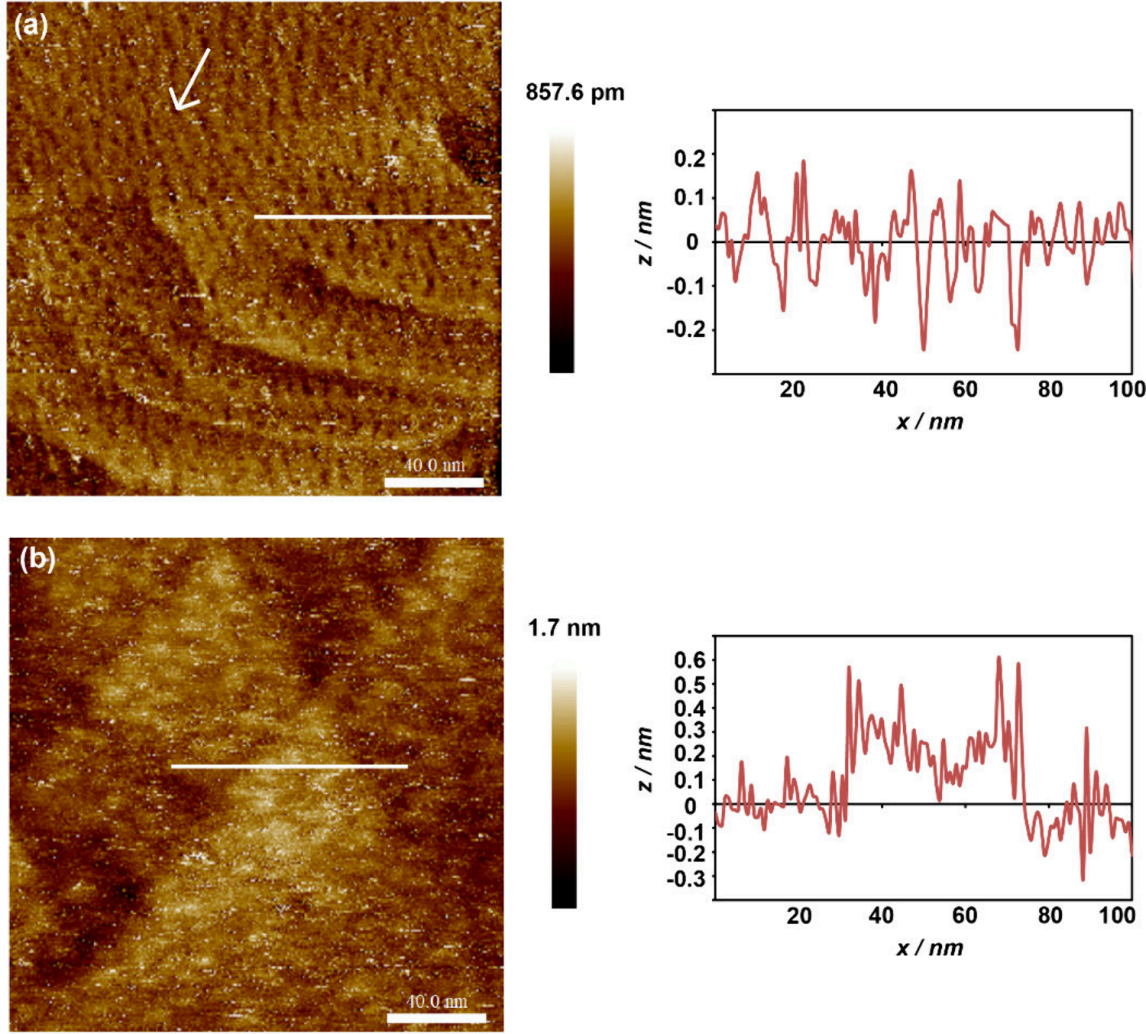
200 nm × 200 nm STM images of films of compound (a) **NOBnDS** (**1**) and (b) **NOPDS** (**2**) prepared by immersion of the Au(111) substrate in a 1 mM solution of the corresponding compound for 24 h. White arrow marks gold vacancies. The height profiles across the selected lines are shown next to the STM images.

The nanoscale surface morphology of **NOBnDS** (**1**), **NOPDS** (**2**), and **NOBPDS** (**3**) films was next investigated by STM under ambient conditions. STM images of **NOBnDS** (**1**) films revealed a high number of gold vacancy islands with a characteristic depth of about 0.24 nm (Figure 7a). Such a morphology has been reported for films of similar adsorbates with an alkyl spacer between the sulfur headgroup and the aromatic moiety.^84,96−99^ To improve the film quality and reduce the number of defects, **NOBnDS** (**1**) films were postannealed at 328 K for 2 h after the room-temperature assembly for 24 h. This procedure resulted in a decrease in the total number of gold vacancy islands, while their average size increased (Figure S42), which is in accordance with the Ostwald ripening law.^100,101^ However, all attempts to achieve molecular resolution were unsuccessful, which suggested that **NOBnDS** (**1**) molecules probably do not form organized films on the Au(111) surface. Films of **NOPDS** (**2**) and **NOBPDS** (**3**), with the disulfide headgroups directly bonded to the aromatic moieties, are characterized by the presence of a high density of irregularly shaped small islands appearing bright in STM images (Figures 7b, S43, and S44). The cross-section profiles suggested that the islands probably represent gold adatoms covered by molecules (Figures S43 and S44). The appearance of gold adatoms agrees with the literature data for similar aromatic thiolates on the gold surface.^98,99,102−105^ As with **NOBnDS** (**1**) films, all attempts to obtain high-resolution STM images of **NOPDS** (**2**) and **NOBPDS** (**3**) films were in vain, even after the postannealing process. In general, aromatic thiolate films on Au(111) usually have poorer quality than aliphatic ones.^93,99,106^ In addition, the formation of organized films of the disulfide-containing nitrosoarenes studied herein could be hindered by their oligomerization on the Au(111) surface.

### Comparison of **NOBnDS** (**1**), **NOPDS** (**2**), and **NOBPDS** (**3**) Films on the Au(111) Surface

Raman spectra of **NOBnDS** (**1**), **NOPDS** (**2**), and **NOBPDS** (**3**) films on the Au(111) substrate revealed the presence of a band typical of the N=N stretch of *E*-azodioxides, which indicated that all three studied nitrosoarenes form *E*-azodioxy oligomers on the Au(111) surface. Ellipsometry-derived thicknesses of disulfide-containing nitrosoarene films on Au(111) showed higher values compared with those of structurally analogous nitro derivatives, which further suggested that azodioxy films are formed in the former case. Nitroso derivative **NOBnDS** (**1**), which contains a methylene group between the phenyl ring and the disulfide headgroup, produced films with the highest values of mean ellipsometric thicknesses at adsorption times of 24 and 48 h, which indicated its tendency to form oligomeric surface structures. The lowest values of the film thicknesses at both adsorption times were observed for **NOPDS** (**2**), which could be explained by the presence of the electron-donating sulfur atom in the *para*-position with respect to the nitroso group diminishing the formation of azodioxides. In **NOBPDS** (**3**), as in **NOPDS** (**2**), the disulfide group is directly attached to the aromatic moiety. Yet, due to the presence of a biphenyl unit, there is no participation of the quinoid resonance structure here, which is manifested by the greater tendency of **NOBPDS** (**3**) to form oligomers on the Au(111) as indicated by larger film thicknesses compared to that for **NOPDS** (**2**). However, compared to **NOBnDS** (**1**) films, **NOBPDS** (**3**) films are composed of a smaller number of monomeric subunits according to ellipsometry measurements. In general, the relatively low values of film thickness could be explained by the possible tilting of the oligomer chains relative to the surface normal and/or their interdigitation. Another possibility is that the adsorbed nitrosoarene molecules are lying flat on top of the initial SAM with both nitrosoaryl ends bonded to the exposed nitroso groups of the bottom SAM via azodioxy bonds, as illustrated in Figure S45. However, this may not be very likely due to the rigid aromatic backbone of the studied nitrosoarenes and increased steric hindrance with molecules adsorbed on the Au(111) surface. For all three studied systems, prolongation of the adsorption time from 24 to 48 h leads to only a relatively small increase in median film thicknesses. Raman and ellipsometry data were corroborated with water contact angle measurements of **NOBnDS** (**1**), **NOPDS** (**2**), and **NOBPDS** (**3**) films formed at adsorption times of 24 and 48 h. The lowest values of water contact angles were observed for **NOPDS** (**2**) films, which is consistent with the expected decreased tendency of **NOPDS** (**2**) to form azodioxides on the Au(111) surface. However, films of nitrosoarenes displayed higher contact angles compared with monolayer films of analogous nitro derivatives, suggesting the formation of azodioxy oligomers on Au(111) to some extent for all three studied compounds. In support of this conclusion, AFM topography images of **NOBnDS** (**1**), **NOPDS** (**2**), and **NOBPDS** (**3**) films revealed the appearance of islands, which is in sharp contrast to the rather homogeneous morphology of the monolayer films of the corresponding nitro derivatives. According to previous studies, such islands were attributed to azodioxides.^51−55^ Large-scale STM images of **NOBnDS** (**1**), **NOPDS** (**2**), and **NOBPDS** (**3**) films revealed morphology typical of aromatic thiolates with (**NOBnDS** (**1**)) or without (**NOPDS** (**2**) and **NOBPDS** (**3**)) an alkyl spacer characterized by the appearance of gold vacancy islands and gold adatoms, respectively. STM measurements indicated the formation of poorly organized films on the Au(111) surface without observing any ordered structure despite numerous attempts and variations in the STM imaging conditions.

## Conclusions

We successfully synthesized three new disulfide-containing nitrosoarenes **NOBnDS** (**1**), **NOPDS** (**2**), and **NOBPDS** (**3**) and investigated the possibility of their self-polymerization through azodioxy bonds on the Au(111) surface. Raman measurements confirmed the presence of *E*-azodioxy groups in the films of **NOBnDS** (**1**), **NOPDS** (**2**), and **NOBPDS** (**3**) on the Au substrates. Raman spectroscopy data were corroborated with ellipsometry measurements, which revealed higher thickness values of nitrosoarene films compared to those of films of structurally analogous nitro derivatives **NO**_**2**_**BnDS** (**1a**), **NO**_**2**_**PDS** (**2b**), and **NO**_**2**_**BPDS** (**3f**), indicating the formation of azodioxy oligomers on the Au(111) surface. The highest tendency for on-surface oligomerization was observed for **NOBnDS** (**1**), which contains a methylene group between the phenyl ring and disulfide functionality. On the contrary, **NOPDS** (**2**) is the least susceptible to the formation of azodioxides on the Au(111) surface, which can be attributed to the substituent electronic effects. According to their obtained thicknesses, azodioxy films probably consist of only a few subunits. The conclusions derived from the Raman and ellipsometry data were additionally supported by water contact angle measurements and AFM images. In contrast to the homogeneous morphology of the nitroarene monolayer films, their nitroso counterparts showed an island-like morphology, previously observed for nitroso/azodioxy films on the Au(111) surface. Prolongation of the adsorption time leads to a slightly higher tendency of oligomerization of disulfide-containing nitrosoarenes on the Au(111) surface. In STM images of nitrosoarene films, no ordered phases were observed, which indicated the formation of poorly organized surface structures in which oligomer chains are possibly interdigitated and substantially tilted relative to the surface normal. This could also explain the relatively low values of the ellipsometry thicknesses of the nitrosoarene films.

The results of the current work revealed the interrelationship between the molecular and surface structural features of the disulfide-containing nitrosoarenes and contributed to a fundamental understanding of the interactions of aromatic C-nitroso compounds at the gold–solution interface. Furthermore, self-polymerization of nitrosoarenes with disulfide functionalities could be used for the relatively simple design of azodioxy thiolate films on gold surfaces with various possible applications, e.g., in the fabrication of electronic devices.

## Supporting Information Available

Synthesis procedures; IR and NMR spectra of all synthesized compounds; Raman spectra of **NOBnDS** (**1**), **NOPDS** (**2**), and **NOBPDS** (**3**) films formed on the Au(111) substrate compared with the spectra of the bare Au(111) substrate; ellipsometry thicknesses, water contact angles, and AFM images of films of disulfide-containing nitro derivatives **NO**_**2**_**BnDS** (**1a**), **NO**_**2**_**PDS** (**2b**) and **NO**_**2**_**BPDS** (**3f**) on the Au(111) surface; AFM images of films of disulfide-containing nitroso derivatives **NOBnDS** (**1**), **NOPDS** (**2**), and **NOBPDS** (**3**) formed on the Au(111) surface after 48 h; comparison of STM images of **NOBnDS** (**1**) films on Au(111) prepared without and with a postannealing step; STM image of films of compound **NOPDS** (**2**) formed on the Au(111) substrate after 24 h; STM image of films of compound **NOBPDS** (**3**) formed on the Au(111) substrate after 24 h; and schematic representation of the self-polymerization of disulfide-containing nitrosoarenes **NOBnDS** (**1**), **NOPDS** (**2**), and **NOBPDS** (**3**) on the Au(111) surface by creating a bridge through azodioxy bonds on top of the initial SAM (PDF)
