# Supplementary material for: Disulfide-Containing Nitrosoarenes: Synthesis and Insights into Their Self-Polymerization on a Gold Surface
Source: Langmuir. 2024 Dec 9;41(5):3066–77. doi: 10.1021/acs.langmuir.4c03274 (PMC11823599; doi:10.1021/acs.langmuir.4c03274)
Supplement: Supplementary file 1 — la4c03274_si_001.pdf [file la4c03274_si_001.pdf]

Disulfide-Containing Nitrosoarenes: Synthesis and Insights into Their Self-Polymerization on a Gold Surface

Laura Nuić, [†] *Ana Senkić* [‖] *Željka Car,* [†] *Ena Asić,* [†] *Nataša Vujičić,* [‖] *Marko Kralj,* [‖] *Ivana Biljan* [†,*]

[†]Department of Chemistry, Faculty of Science, University of Zagreb, Horvatovac 102A, HR-10000 Zagreb, Croatia

[‖]Center for Advanced Laser Techniques, Institute of Physics, Bijenička cesta 46, HR-10000 Zagreb, Croatia

[*]**Corresponding Author.**

E-mail address: ibiljan@chem.pmf.hr (I. Biljan).

Supporting Information

Table of contents

1. **Synthesis** 3
2. **IR spectra** 8
3. **NMR spectra** 14
4. **Raman spectra** 27
5. **Ellipsometry and contact angle measurements** 28
6. **AFM images** 29
7. **STM images** 30

1. Synthesis

Synthesis of bis(4-nitrobenzyl) disulfide (**1a**)

Commercially available 4-nitrobenzyl bromide (2.31 mmol) was dissolved in DMSO (5 mL). The solution of $Na_2S_2O_3 \times 5H_2O$ (2.31 mmol) in distilled H_2O (0.5 mL) was added. The reaction mixture was stirred for 45 min at 65 °C. After completion of the reaction, H_2O was added and extracted with DCM. The organic layer was washed with distilled H_2O (3×) and dried over anhydrous Na_2SO_4. The obtained mixture was left in a small amount of DCM in the refrigerator for 24 h, resulting in precipitating the product. The precipitate was filtered through a Büchner funnel to afford a pure product as a white solid with a yield of 56 %.

^{1}H (400 MHz, $CDCl_3$) δ/ppm: 8.20 (d, 2H, J = 8.8 Hz); 7.39 (d, 2H, J = 8.8 Hz); 3.70 (s, 2H). ^{13}C (100 MHz, $CDCl_3$) δ/ppm: 147.6 (C–NO_2); 145.0 (C); 130.4 (CH–Ph); 124.1 (CH–Ph); 42.5 (CH_2). IR (ATR) $\tilde{\upsilon}$/cm^{-1}: 3078 (=C–H); 1609 (C=C); 1537 (NO_2 as); 1344 (NO_2 s); 525 (S-S). MS (ESI) m/z: Calculated [M] for $C_{14}H_{12}N_2O_4S_2$ 336.02385 and found $[M\text{-}H]^-$ 335.0172.

Synthesis of 4-nitrophenyl thiol (**2a**)

Commercially available 1-iodo-4-nitrobenzene (2.001 mmol) was dissolved in DMF (6 mL). S (6.02 mmol) and K_2CO_3 (4.02 mmol) were added. The reaction mixture was stirred for 4 h at 75 °C in an argon atmosphere. After 4 h, dioxane (6 mL), PPh_3 (6.02 mmol), H_2O (6 mL) and 2M HCl (3 mL) were added to the reaction mixture. The reaction mixture was then stirred for 24 h at 45 °C in an argon atmosphere. After the completion of the reaction, 10 % HCl was added to the reaction mixture and extracted with EtOAc. The organic layer was washed with a saturated aqueous solution of $NaHCO_3$ (4×). The aqueous layer was acidified with concentrated HCl (until colorless) and extracted with EtOAc. The organic layer was washed then with Brine solution and dried over anhydrous Na_2SO_4. The solvent was evaporated to afford a pure product as a pale-yellow solid with a yield of 54 %.

^{1}H (400 MHz, $CDCl_3$) δ/ppm: 8.09 (d, 2H, J = 8.9 Hz); 7.36 (d, 2H, J = 8.9 Hz); 3.77 (s, 1H). ^{13}C (100 MHz, $CDCl_3$) δ/ppm: 145.9 (C–NO_2); 142.2 (C); 128.7 (CH–Ph); 124.6 (CH–Ph). IR (ATR) $\tilde{\upsilon}$/cm^{-1}: 3098 (=C–H); 2547 (S-H); 1575 (C=C); 1537 (NO_2 as); 1329 (NO_2 s). MS (ESI) m/z: Calculated [M] for $C_6H_5NO_2S$ 155.0041 and found $[M\text{-}H]^-$ 153.9976.

Synthesis of bis(4-nitrophenyl) disulfide (**2b**)

The synthesized compound **2a** (1 mmol) was dissolved in EtOAc (3 mL). NaI (0.01 mmol) and 30 % H_2O_2 (1 mmol) were added. The reaction mixture was stirred for 45 min at room temperature. After completion of the reaction, the saturated aqueous solution of $Na_2S_2O_3$ (15 mL) was added and the reaction mixture was extracted with EtOAc (3×). The organic layer was washed with a saturated aqueous solution of NaCl and dried over anhydrous Na_2SO_4. The solvent was evaporated to afford a pure product as a pale-yellow solid with a yield of 87 %.

^{1}H (400 MHz, $CDCl_3$) δ/ppm: 8.19 (d, 4H, J = 9.0 Hz); 7.62 (d, 4H, J = 9.0 Hz). ^{13}C (100 MHz, $CDCl_3$) δ/ppm: 147.3 (C–NO_2); 144.4 (C); 126.7 (CH–Ph); 124.8 (CH-Ph). IR (ATR) $\tilde{\upsilon}$/cm^{-1}: 3095 (=C–H); 1595 (C=C); 1504 (NO_2 as); 1337 (NO_2 s); 554 (S-S). MS (ESI) m/z: Calculated [M] for $C_{12}H_8N_2O_4S_2$ 307.9926 and found [M-H]$^-$ 306.9857.

Synthesis of 4-nitrophenyl boronic acid (**3a**)

Commercially available 4-nitroaniline (3.62 mmol) was dissolved in MeOH (8 mL). Concentrated HCl (1.85 mL) and distilled H_2O (2 mL) were added. After stirring for 3 min at 0 °C, a solution of $NaNO_2$ (4.35 mmol) in distilled H_2O (1 mL) was added. The reaction mixture was stirred for 75 min at 0 °C. After 75 min, a $B_2(OH)_4$ (0.011 mol) was added. The reaction mixture was then stirred for 90 min at room temperature. After completion of the reaction, EtOAc and 2 M Na_2CO_3 solution were added to the reaction mixture until pH=8. The organic layer was extracted with Na_2CO_3 solution (2×). The aqueous layer was acidified with concentrated HCl to pH=1 and extracted with EtOAc. The organic layer was then washed with Brine solution and dried over anhydrous Na_2SO_4. The solvent was evaporated to afford a pure product as a pale-yellow solid with a yield of 72 %.

^{1}H (400 MHz, DMSO-d_6) δ/ppm: 8.50 (s, 2H); 8.19 (d, 2H, J = 8.6 Hz); 8.03 (d, 2H, J = 8.6 Hz). ^{13}C (100 MHz, DMSO-d_6) δ/ppm: 149.7 (C–NO_2); 143.1 (C); 136.2 (CH–Ph); 123.0 (CH-Ph). IR (ATR) $\tilde{\upsilon}$/cm^{-1}: 3500 (B-OH); 3104 (=C–H); 1595 (C=C); 1499 (NO_2 as); 1335 (NO_2 s); 1318 (B-O). MS (ESI) m/z: Calculated [M] for $C_6H_6NO_4B$ 167.0389 and found [M-H]$^-$ 166.0326.

Synthesis of 4'-nitrobiphenyl-4-ol (**3b**)

Commercially available 4-bromophenol (1.81 mmol) was dissolved in toluene (8 mL). Under a nitrogen atmosphere $Pd(PPh_3)_4$ (0.055 mmol), 2 M Na_2CO_3 solution (3 mL), followed by synthesized compound **3a** (1.81 mmol) was added. The reaction mixture was stirred for 3 h at 80 °C. After 3 h, EtOAc was added and the reaction mixture was extracted with a saturated aqueous solution of NaCl (2×). The organic layer was dried over anhydrous Na_2SO_4. The desiccant is filtered, and the solvent is evaporated on a rotary evaporator. After the evaporation of the solvent, the obtained solid was purified by column chromatography eluting with DCM to afford a pure product as a yellow-orange solid with a yield of 28 %.

^{1}H (400 MHz, DMSO-d_6) δ/ppm: 9.88 (s, 1H); 8.25 (d, 2H, J = 8.9 Hz); 7.88 (d, 2H, J = 8.9 Hz); 7.66 (d, 2H, J = 8.7 Hz); 6.91 (d, 2H, J = 8.7 Hz). ^{13}C (100 MHz, DMSO-d_6) δ/ppm: 159.6 (C–OH); 147.6 (C–NO_2); 146.7 (C); 129.5 (CH–Ph); 129.2 (C); 127.6 (CH-Ph); 125.0 (CH-Ph); 117.0 (CH-Ph). IR (ATR) $\tilde{\nu}$/cm^{-1}: 3417 (O-H); 2923 (=C–H); 1591 (C=C); 1505 (NO_2 as); 1333 (NO_2 s). MS (ESI) m/z: Calculated [M] for $C_{12}H_9NO_3$ 215.0582 and found $[M-H]^-$ 214.0524.

Synthsis of 4'-nitrobiphenyl-4-O-(N,N-dimethylthiocarbamate) (**3c**)

The synthesized compound **3b** (0.465 mmol) was dissolved in dry DMF (3 mL). 90 % NaH (0.75 mmol) was then slowly added at 0 °C in a nitrogen atmosphere. After all the NaH was added, the reaction mixture was stirred for 12 h at 0 °C. After 12 h, $ClOSCNMe_2$ (0.697 mmol) was added and the reaction mixture was stirred for 12 h at 60 °C. After completion of the reaction, the solvent was evaporated washing it with DCM. After the evaporation of the solvent, the obtained solid was purified by column chromatography eluting with DCM to afford a pure product as a yellow solid with a yield of 46 %.

^{1}H (400 MHz, $CDCl_3$) δ/ppm: 8.30 (d, 2H, J = 8.9 Hz); 7.74 (d, 2H, J = 8.9 Hz); 7.65 (d, 2H, J = 8.7 Hz); 7.21 (d, 2H, J = 8.7 Hz); 3.49 (s, 1H); 3.39 (s, 3H). ^{13}C (100 MHz, $CDCl_3$) δ/ppm: 187.7 (C=S); 155.0 (C); 147.4 (C); 147.1 (C); 136.7 (C); 128.6 (CH-Ph); 128.1 (CH-Ph); 124.5 (CH-Ph); 124.0 (CH-Ph); 43.7 (CH_3); 39.2 (CH_3). IR (ATR) $\tilde{\nu}$/cm^{-1}: 1593 (C=C); 1516 (NO_2 as); 1337 (NO_2 s). MS (ESI) m/z: Calculated [M] for $C_{15}H_{14}SN_2O_3$ 302.0725 and found $[M+H]^+$ 303.0816.

Synthesis of 4'-nitrobiphenyl-4-S-(N,N-dimethylthiocarbamate) (**3d**)

The preparation of compound **3d** was optimized using microwave assisted synthesis (Table S1). Experiments were performed with or without the solvent (DMA). In the scale-up reaction compound **3c** (1.65 mmol) was dissolved in DMA (5 mL). The reaction mixture was heated to 250 °C for a hold time of 35 min at 300 psi and 300 W. After reaction completion the solvent was evaporated. The obtained solid was purified by column chromatography eluting with DCM to afford a pure product as a pale-yellow solid with a yield of 59 %.

^{1}H (400 MHz, $CDCl_3$) δ/ppm: 8.30 (d, 2H, J = 8.9 Hz); 7.73 (d, 2H, J = 8.9 Hz); 7.62 (s, 4H); 3.12 (bs, 3H); 3.05 (bs, 3H). ^{13}C (100 MHz, $CDCl_3$) δ/ppm: 166.7 (C-S); 147.6 (C); 147.1 (C); 139.8 (C); 136.6 (C); 130.3 (CH-Ph); 128.2 (CH-Ph); 128.1 (CH-Ph); 124.5 (CH-Ph); 37.3 (CH_3). IR (ATR) $\tilde{\upsilon}$/cm^{-1}: 2931 (=C-H); 1597 (C=C); 1511 (NO_2 as); 1339 (NO_2 s). MS (ESI) m/z: Calculated [M] for C15H14SN2O3 302.0725 and found [M+H]+ 303.807.

Table S1. Optimization of microwave conditions for the preparation of **3d**

**3d**					
experiment	reactant *m* /mg	solvent/mL	hold time/min	temperature/°C	yield/%
1	60	/	60	230	33
2	60	/	90	220	31
3	50	/	45	230	33
4	50	DMA/0.5	20	230	28
5	50	DMA/0.5	40	250	36
6	50	DMA/0.5	35	250	61
7	50	DMA/0.5	45	250	62
8	50	DMA/0.5	35	270	22
9	50	DMA/0.5	25	250	52
10	50	DMA/0.25	35	250	52
11	50	DMA/0.75	35	250	59
12	250	DMA/2.5	35	250	54
13	500	DMA/5	35	250	59

Synthesis of 4'-nitrobiphenyl-4-thiol (**3e**)

The synthesized compound **3d** (0.99 mmol) was dissolved in MeOH (10 mL). KOH (11.9 mmol) was added under the nitrogen atmosphere. The reaction mixture was stirred for 6 h at 80 °C. After completion of the reaction, the reaction mixture was cooled and 2 M HCl was added drop by drop until pH=2. The obtained solid was filtered through a Büchner funnel and washed with distilled H_2O. After the solvent was evaporated, the obtained solid was purified by column chromatography eluting with DCM to afford a pure product as a pale-yellow solid with a yield of 89 %.

^1H (400 MHz, $CDCl_3$) δ/ppm: 8.29 (d, 2H, J = 8.8 Hz); 7.70 (d, 2H, J = 8.8 Hz); 7.50 (d, 2H, J = 8.8 Hz); 7.38 (d, 2H, J = 8.8 Hz); 3.56 (s, 1H). ^{13}C (100 MHz, $CDCl_3$) δ/ppm: 147.3 (C-NO_2); 147.0 (C); 136.2 (C); 133.1 (C); 130.0 (CH-Ph); 128.3 (CH-Ph); 127.7 (CH-Ph); 124.5 (CH-Ph). IR (ATR) $\tilde{\upsilon}$/cm^{-1}: 2924 (=C-H); 2571 (S-H); 1594 (C=C); 1504 (NO_2 as); 1338 (NO_2 s). MS (ESI) m/z: Calculated [M] for $C_{12}H_9SNO_2$ 231.0354 and found $[M+H]^+$ 230.0286.

1,2-bis(4'-nitro-[1,1'-biphenyl]-4-yl)disulfane (**3f**)

The synthesized compound **3e** (1.3 mmol) was dissolved in EtOAc (7.5 mL). NaI (0.013 mmol) and 30 % H_2O_2 (150 µL) were added. The reaction mixture was stirred for 35 min at room temperature. After completion of the reaction, the saturated aqueous solution of $Na_2S_2O_3$ (15 mL) was added and the reaction mixture was extracted with EtOAc (3×). The organic layer was washed with a saturated aqueous solution of NaCl and dried over anhydrous Na_2SO_4. The solvent was evaporated to afford a pure product as a yellow solid with a yield of 96 %.

^1H (400 MHz, $CDCl_3$) δ/ppm: 8.29 (d, 4H, J = 8.8 Hz); 7.70 (d, 4H, J = 8.8 Hz); 7.65 (d, 4H, J = 8.6 Hz); 7.59 (d, 4H, J = 8.6 Hz). ^{13}C (100 MHz, $CDCl_3$) δ/ppm: 147.6 (C-NO2); 146.8 (C); 138.2 (C); 138.1 (C-S); 128.4 (CH-Ph); 128.2 (CH-Ph); 127.9 (CH-Ph); 124.6 (CH-Ph). IR (ATR) $\tilde{\upsilon}$/cm^{-1}: 2924 (=C–H); 1594 (C=C); 1511 (NO_2 as); 1338 (NO_2 s); 523 (S-S). MS (ESI) m/z: Calculated [M] for $C_{24}H_{16}N_2O_4S_2$ 460.0552 and found $[M-H]^-$ 459.0504.

2. IR spectra

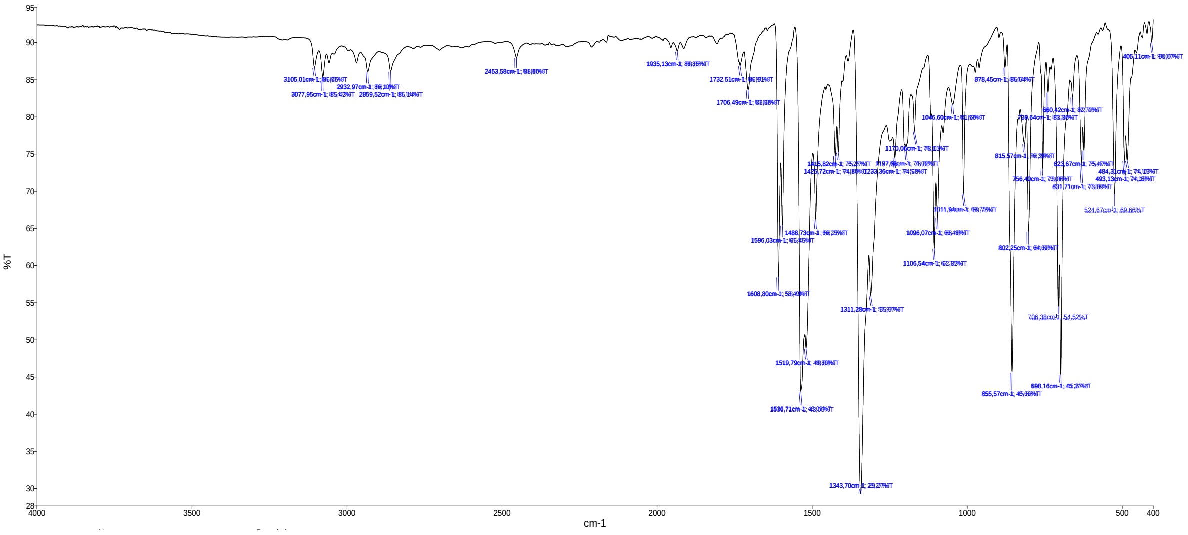

Figure S1. IR (ATR) spectrum of bis(4-nitrobenzyl) disulfide, **NO_2BnDS** (**1a**).

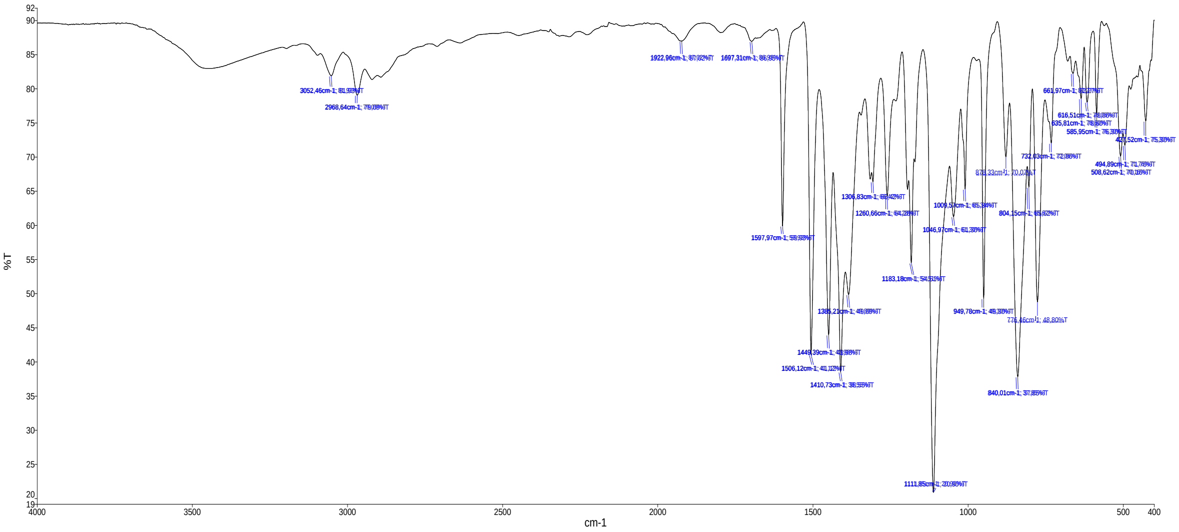

Figure S2. IR (ATR) spectrum of bis(4-nitrosobenzyl) disulfide, **NOBnDS** (**1**).

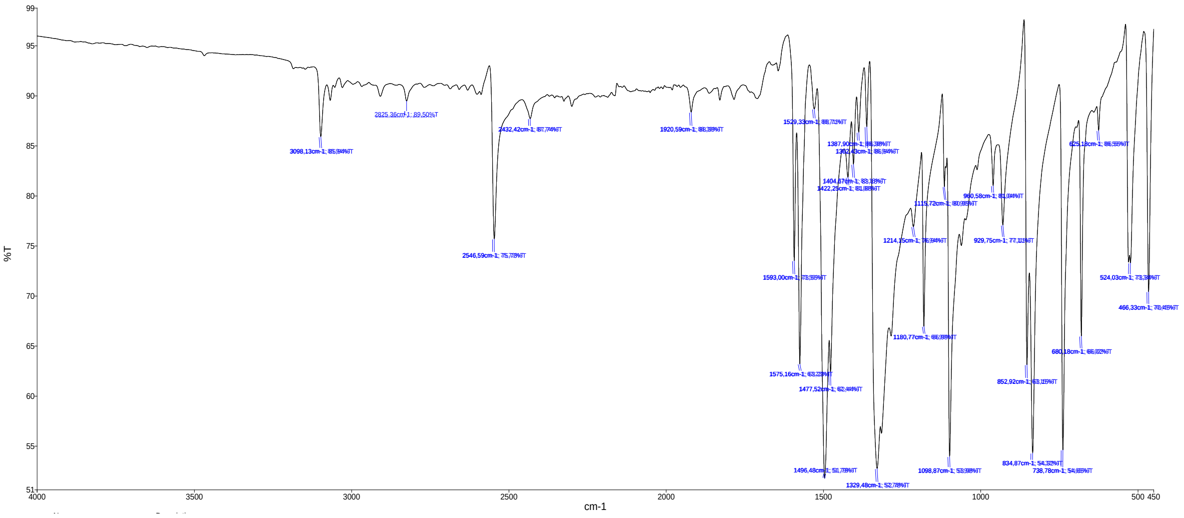

Figure S3. IR (ATR) spectrum of 4-nitrophenyl thiol (**2a**).

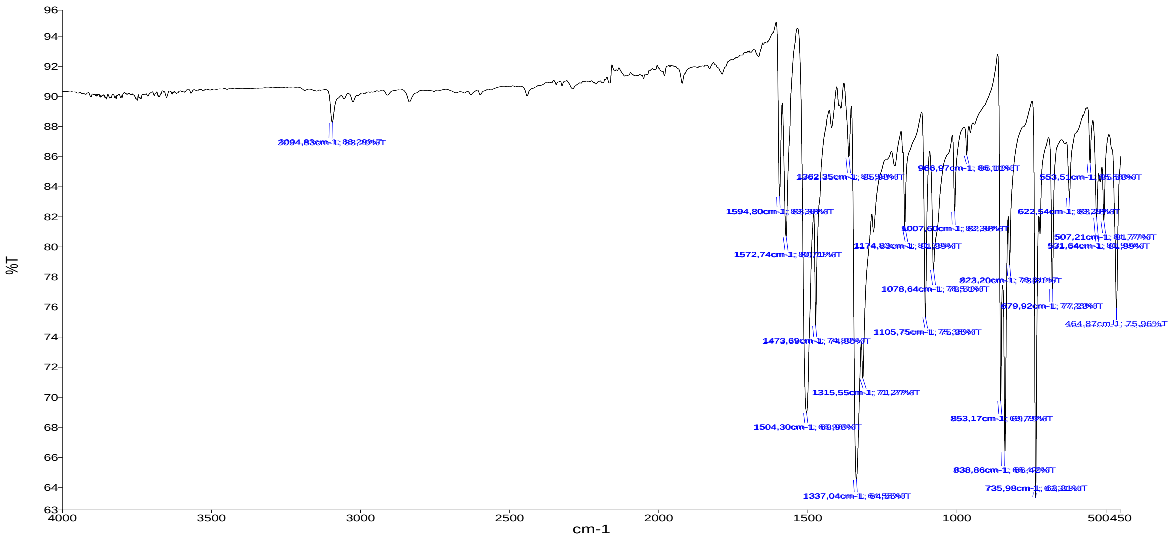

Figure S4. IR (ATR) spectrum of bis(4-nitrophenyl) disulfide, **NO_2PDS** (**2b**).

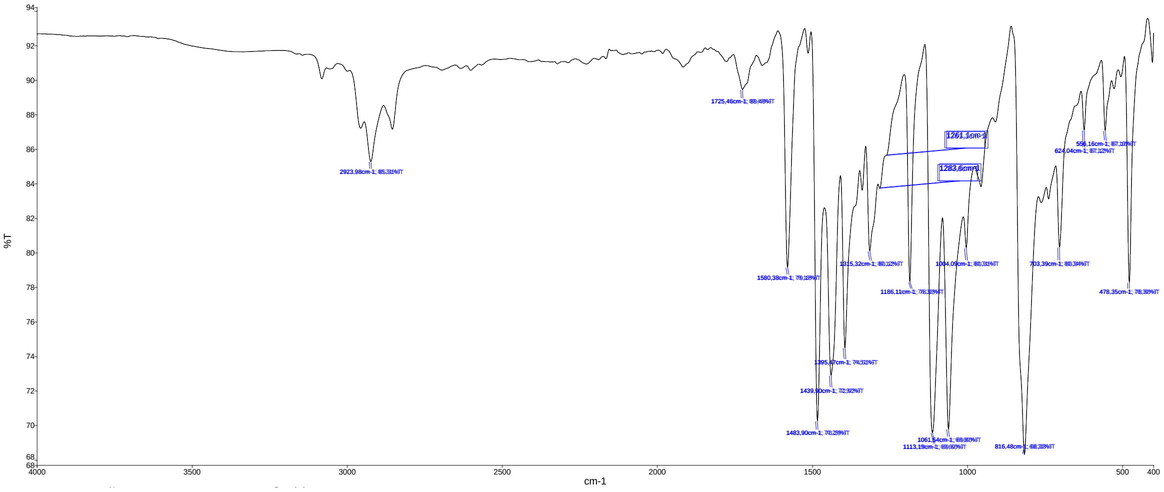

Figure S5. IR (ATR) spectrum of bis(4-nitrosophenyl) disulfide, **NOPDS** (**2**).

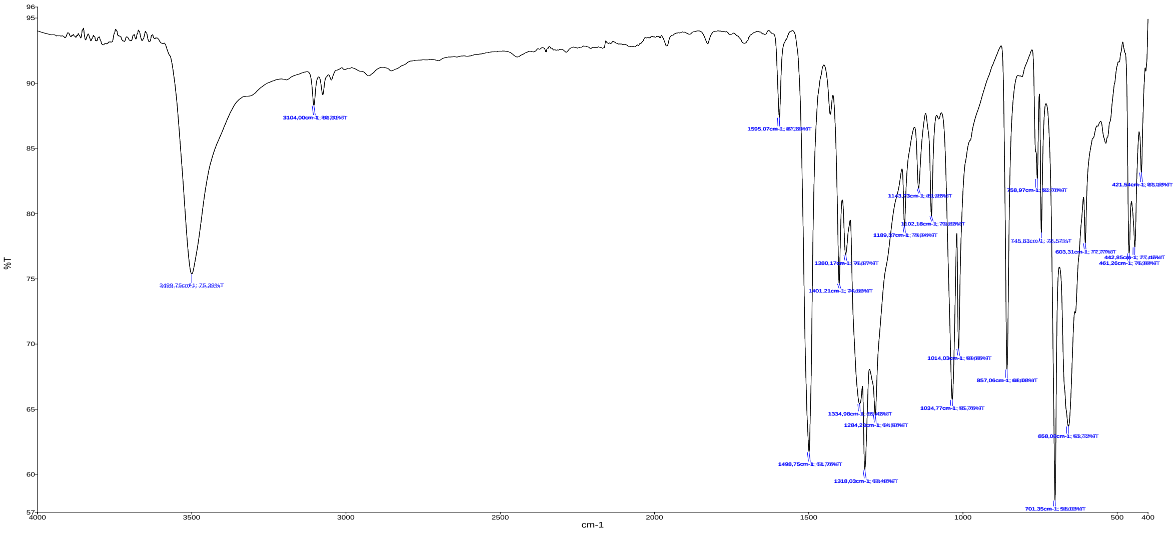

Figure S6. IR (ATR) spectrum of 4-nitrophenyl boronic acid (**3a**).

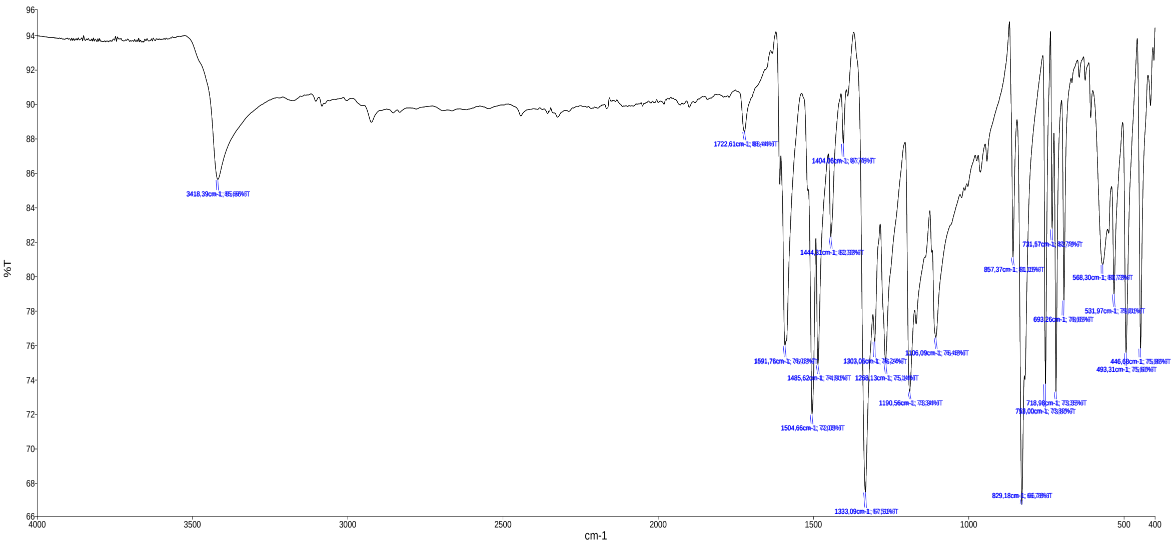

Figure S7. IR (ATR) spectrum of 4'-nitrobiphenyl-4-ol (**3b**).

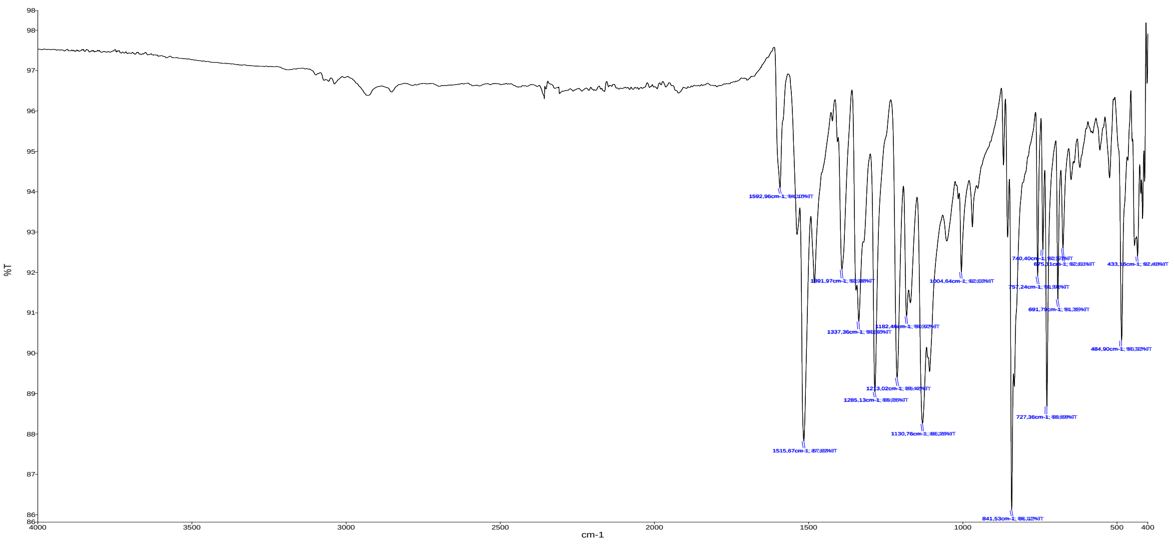

Figure S8. IR (ATR) spectrum of 4'-nitrobiphenyl-4-O-(N,N-dimethylthiocarbamate) (**3c**).

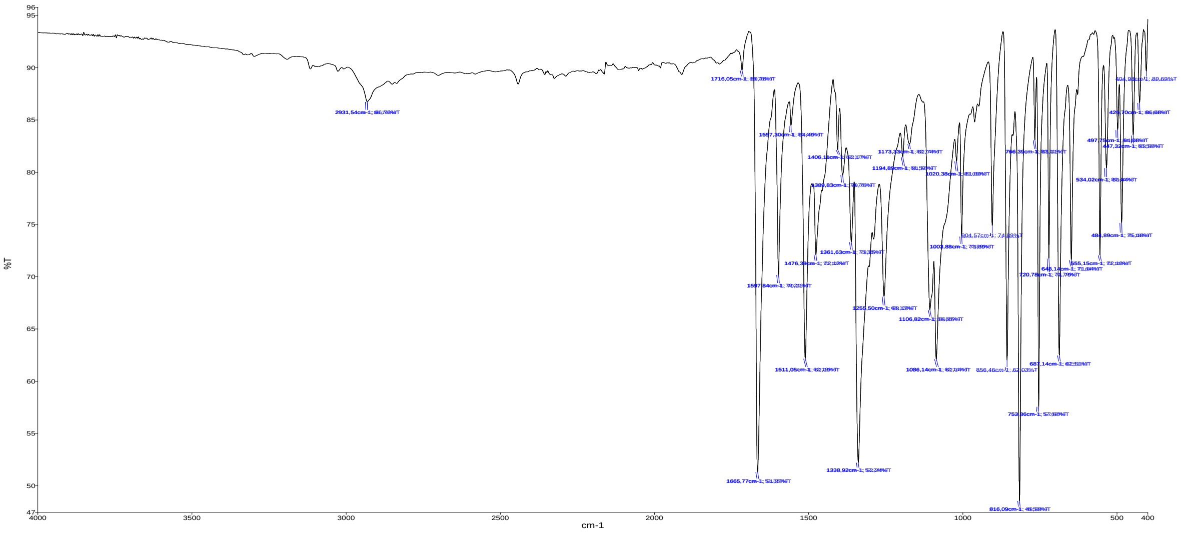

Figure S9. IR (ATR) spectrum of 4'-nitrobiphenyl-4-S-(N,N-dimethylthiocarbamate) (**3d**).

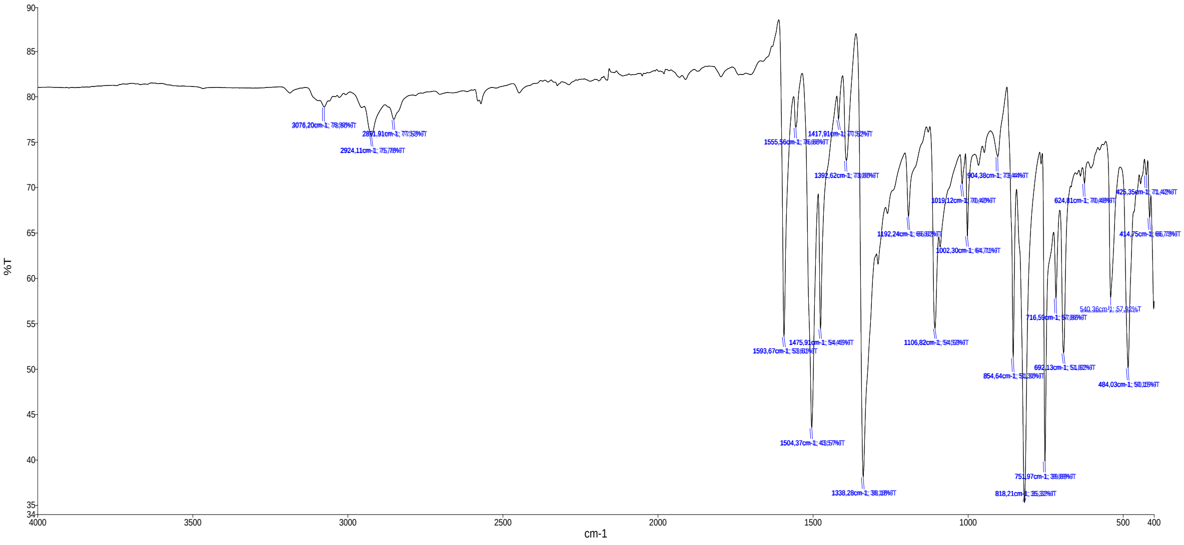

Figure S10. IR (ATR) spectrum of 4'-nitrobiphenyl-4-thiol (**3e**).

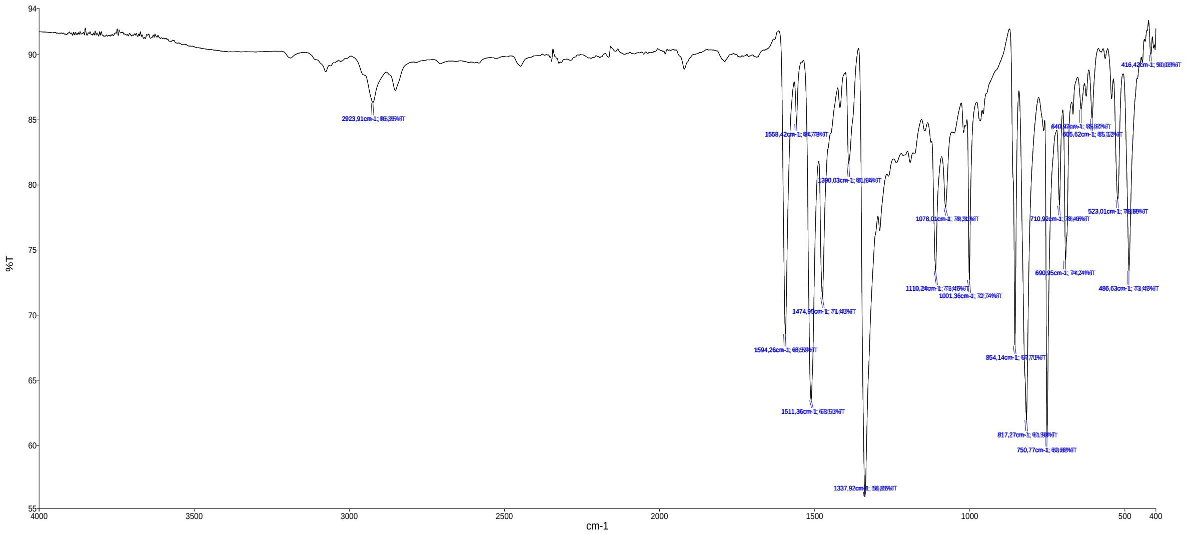

Figure S11. IR (ATR) spectrum of 1,2-bis(4'-nitro-[1,1’-biphenyl]-4-yl)disulfane, **NO_2BPDS** (**3f**).

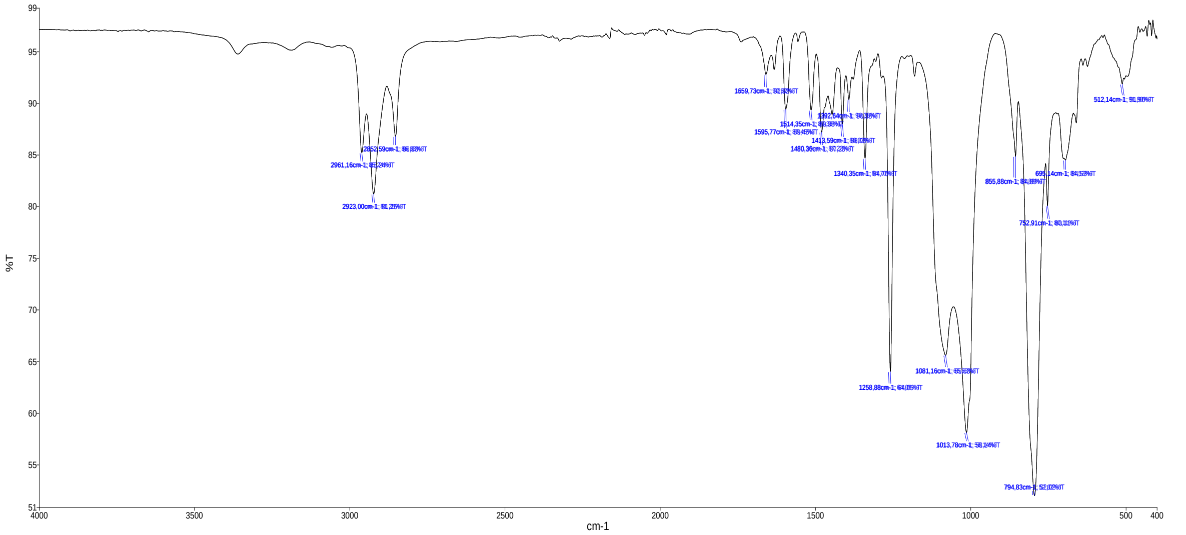

Figure S12. IR (ATR) spectrum of 1,2-bis(4'-nitroso-[1,1’-biphenyl]-4-yl)disulfane, **NOBPDS** (**3**).

3. NMR spectra

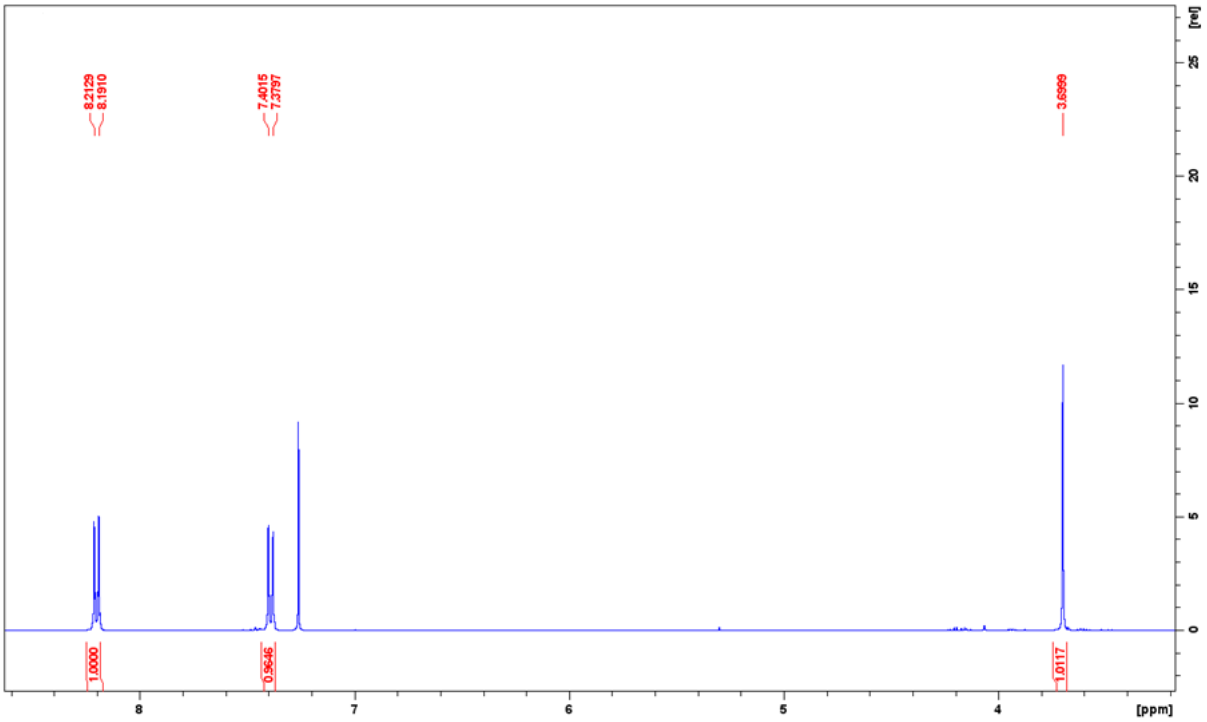

Figure S13. ^{1}H NMR spectrum of bis(4-nitrobenzyl) disulfide, **NO2BnDS** (**1a**) in $CDCl_3$.

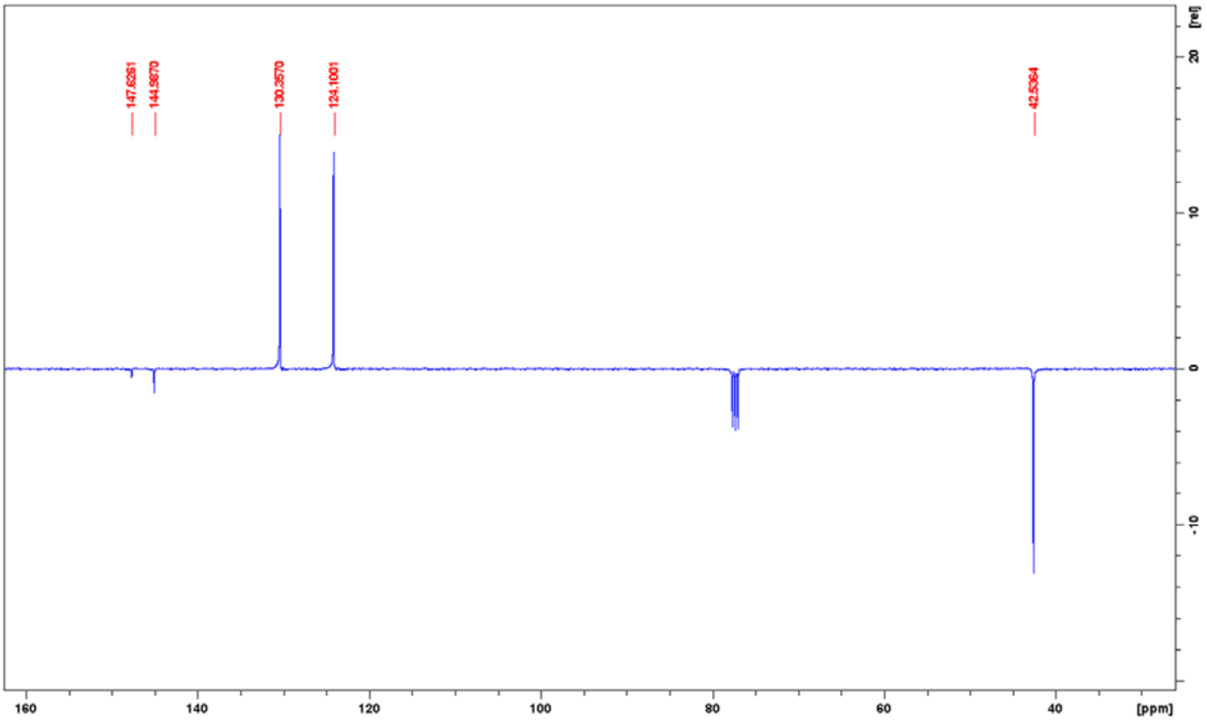

Figure S14. ^{13}C DEPT-Q NMR spectrum of bis(4-nitrobenzyl) disulfide, **NO2BnDS** (**1a**) in $CDCl_3$.

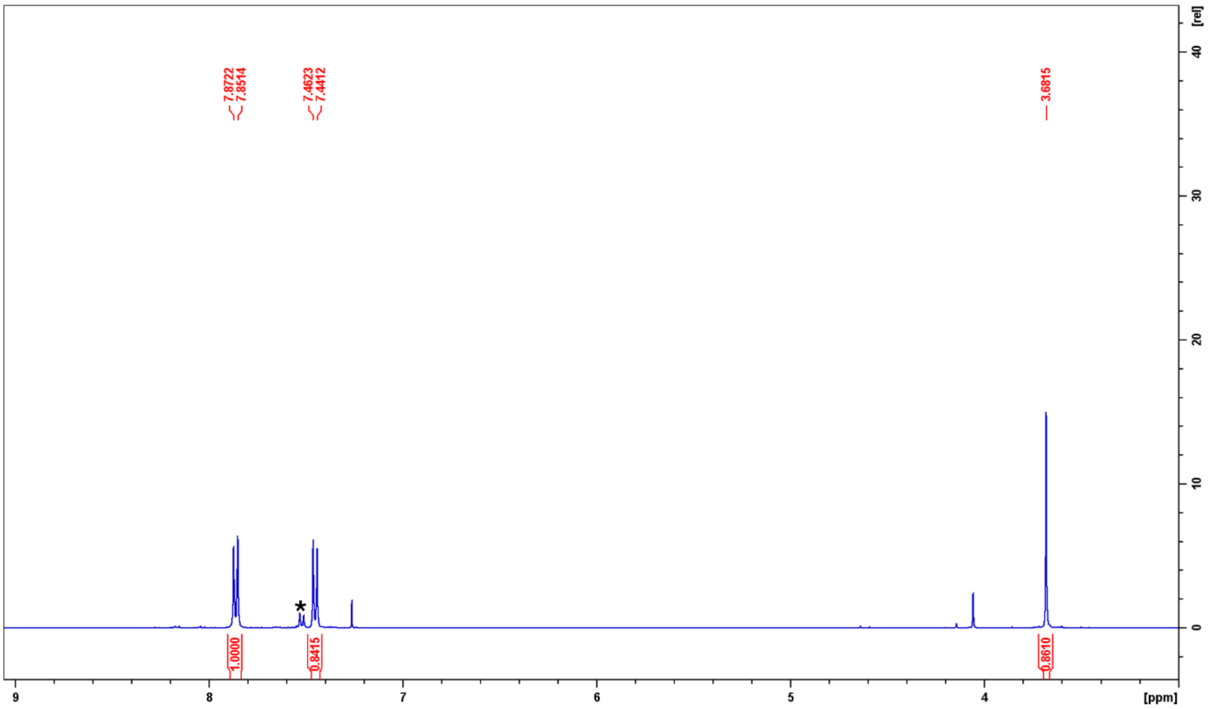

Figure S15. ^{1}H NMR spectrum of bis(4-nitrosobenzyl) disulfide, **NOBnDS** (**1**) in $CDCl_3$. Signal at 7.25 ppm labeled with * corresponds to an unidentified impurity.

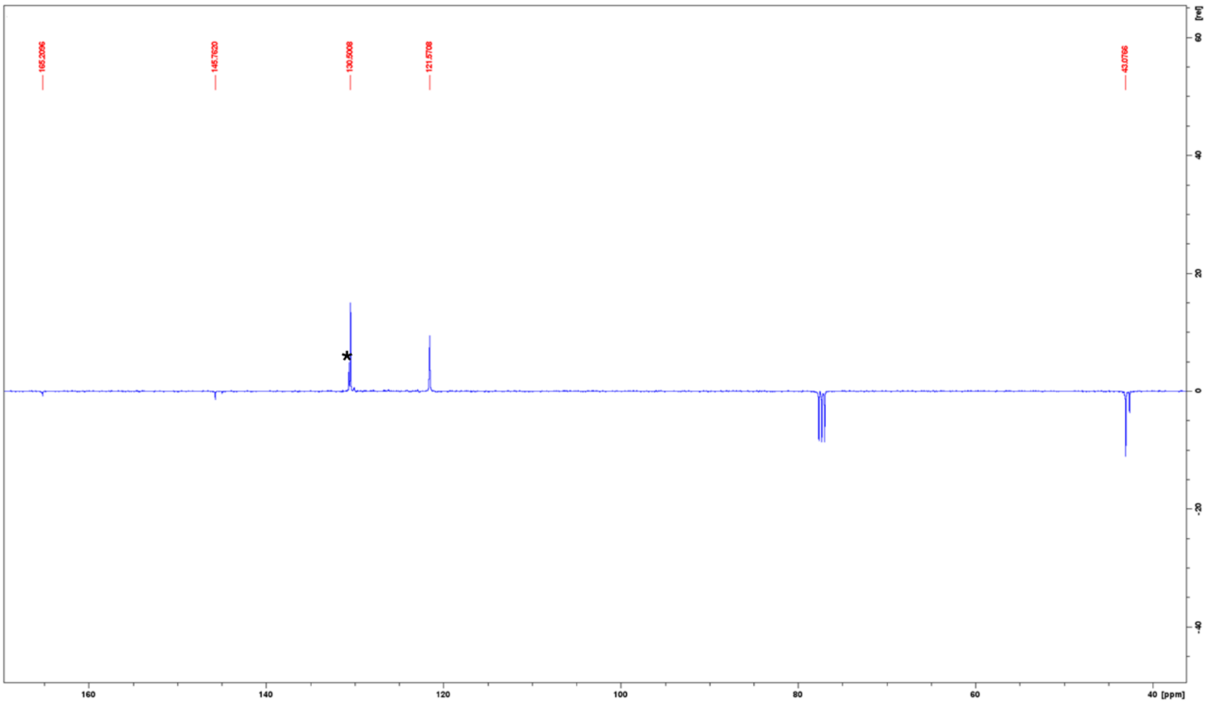

Figure S16. ^{13}C DEPT-Q NMR spectrum of bis(4-nitrosobenzyl) disulfide, **NOBnDS** (**1**) in $CDCl_3$. Signal at 130.7 ppm labeled with * corresponds to an unidentified impurity.

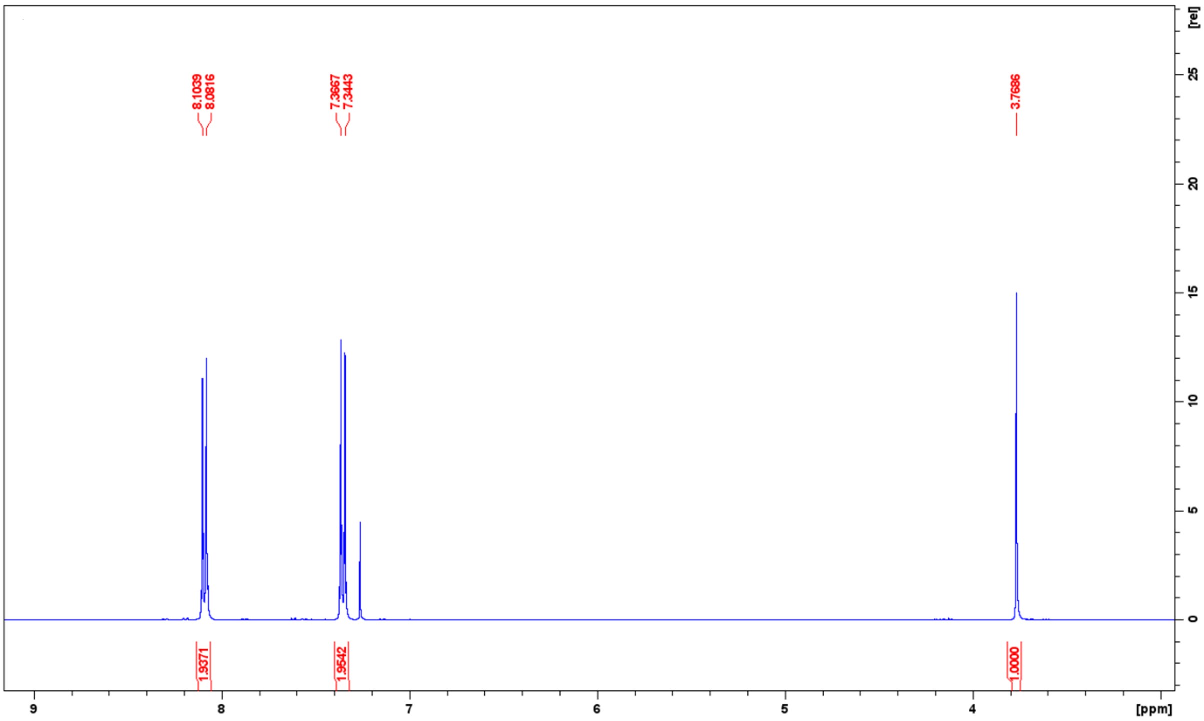

Figure S17. ^{1}H NMR spectrum of 4-nitrophenyl thiol (**2a**) in $CDCl_3$.

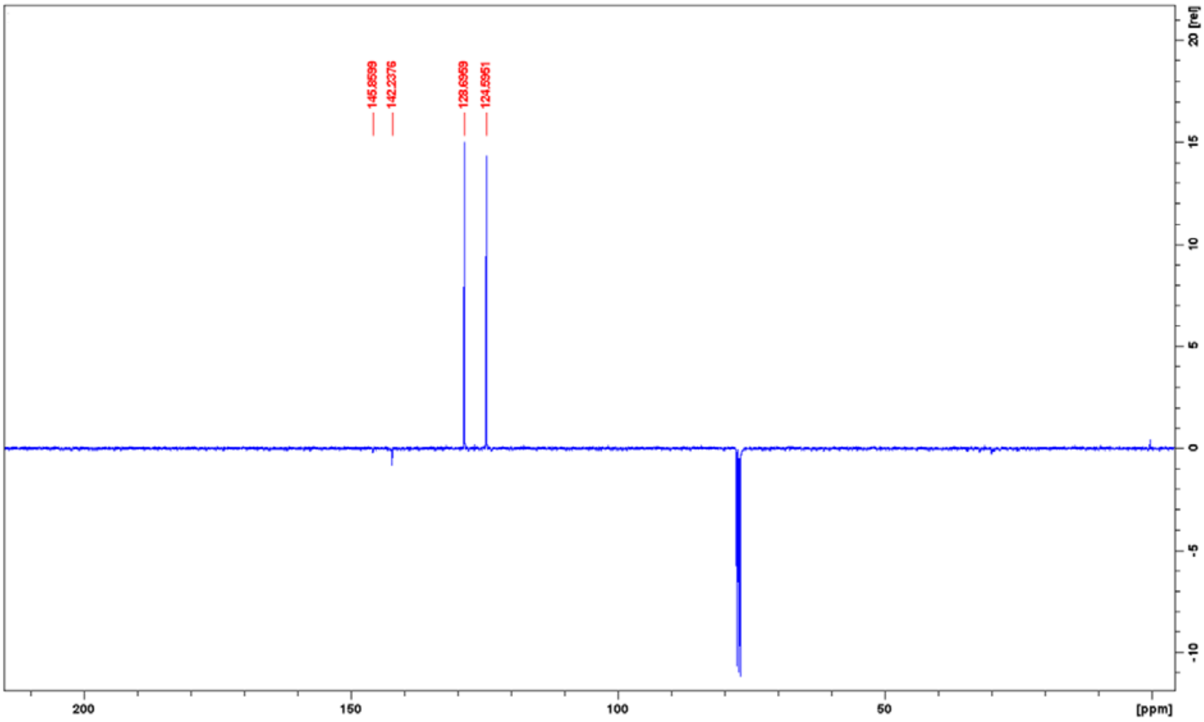

Figure S18. ^{13}C DEPT-Q NMR spectrum of 4-nitrophenyl thiol (**2a**) in $CDCl_3$.

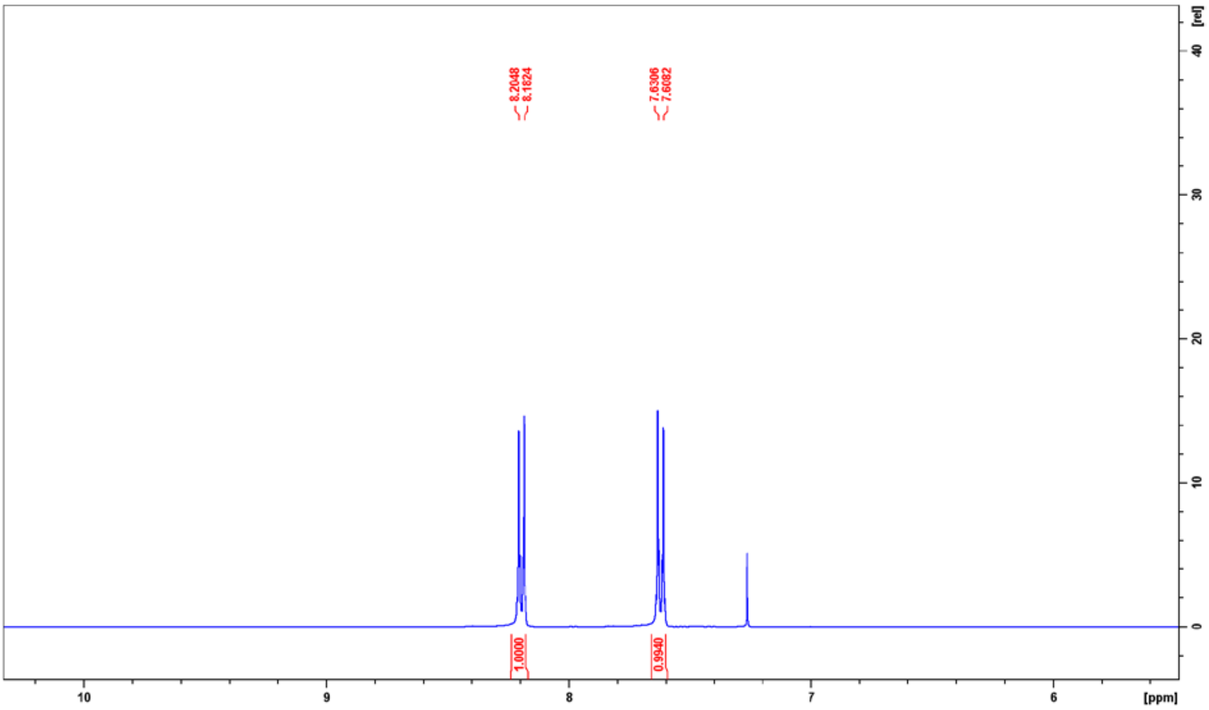

Figure S19. ^{1}H NMR spectrum of bis(4-nitrophenyl) disulfide, **NO_2PDS** (**2b**) in $CDCl_3$.

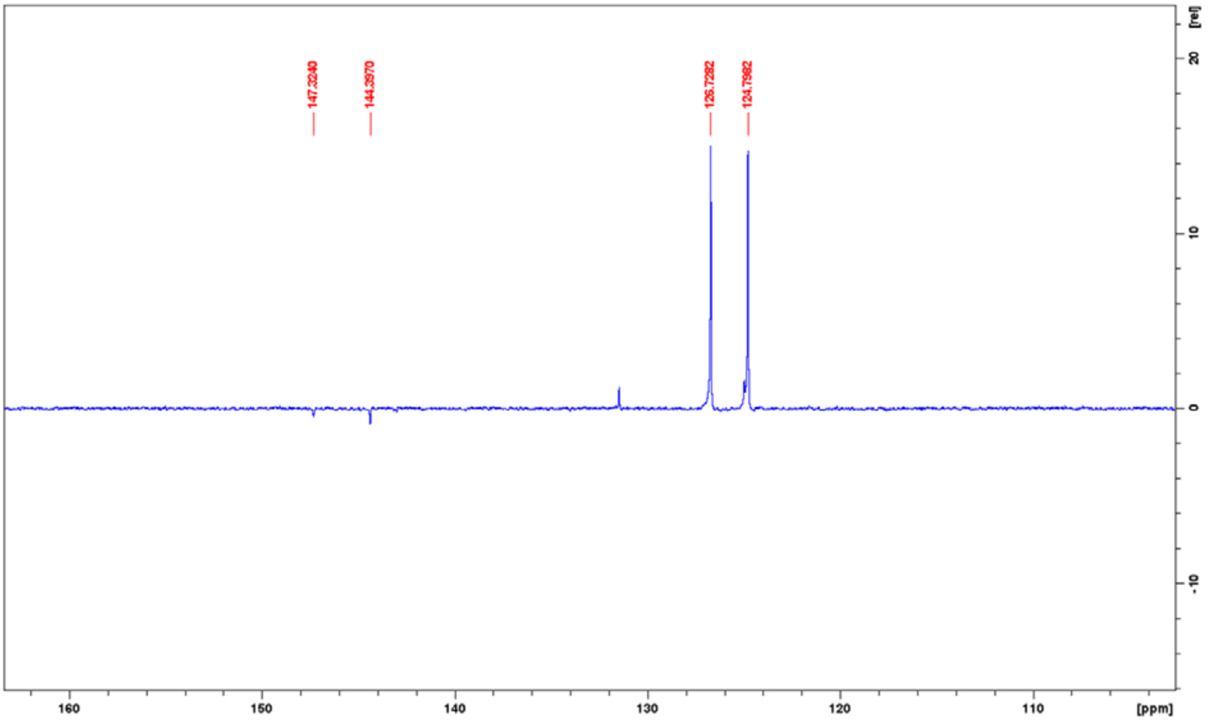

Figure S20. ^{13}C DEPT-Q NMR spectrum of bis(4-nitrophenyl) disulfide, **NO_2PDS** (**2b**) in $CDCl_3$.

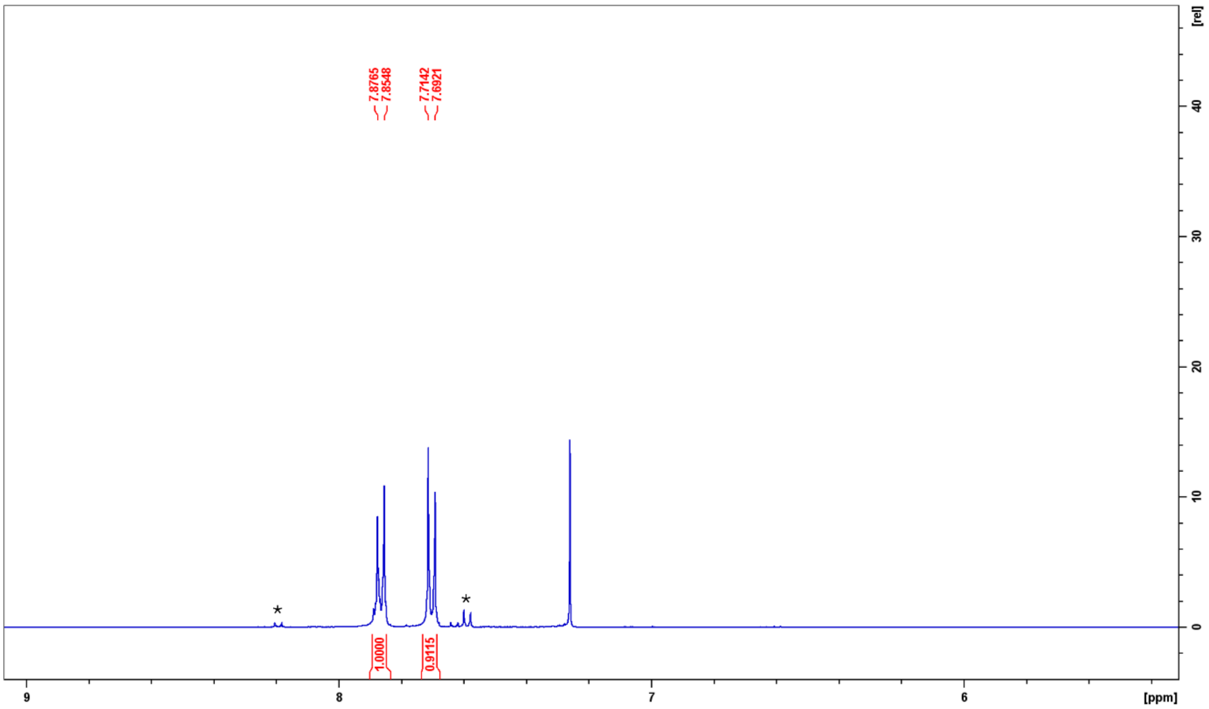

Figure S21. ^{1}H NMR spectrum of bis(4-nitrosophenyl) disulfide, **NOPDS** (**2**) in $CDCl_3$. Signals of impurity (**NO**$_2$**PDS** (**2b**)) at 8.19 ppm and 7.59 ppm are labeled with *.

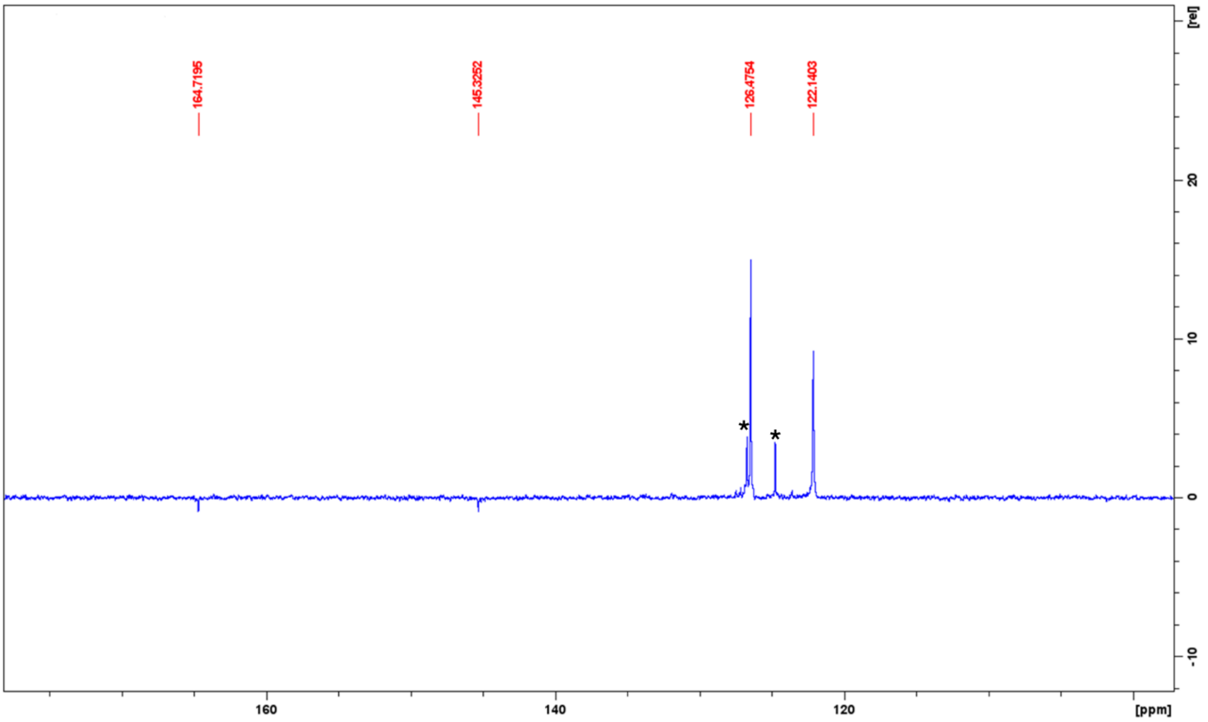

Figure S22. ^{13}C DEPT-Q NMR spectrum of bis(4-nitrosophenyl) disulfide, **NOPDS** (**2**) in $CDCl_3$. Signals of impurity (**NO**$_2$**PDS** (**2b**)) at 126.7 ppm and 124.8 ppm are labeled with *.

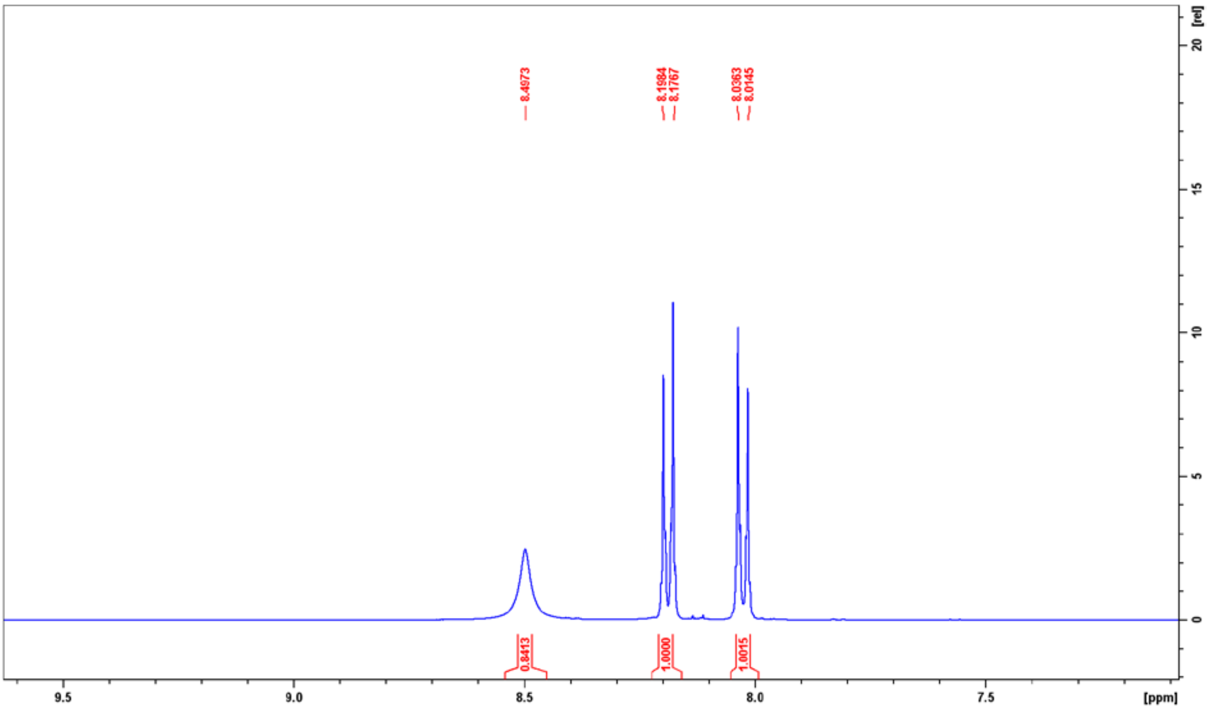

Figure S23. ^{1}H NMR spectrum of 4-nitrophenyl boronic acid (**3a**) in DMSO-d_6.

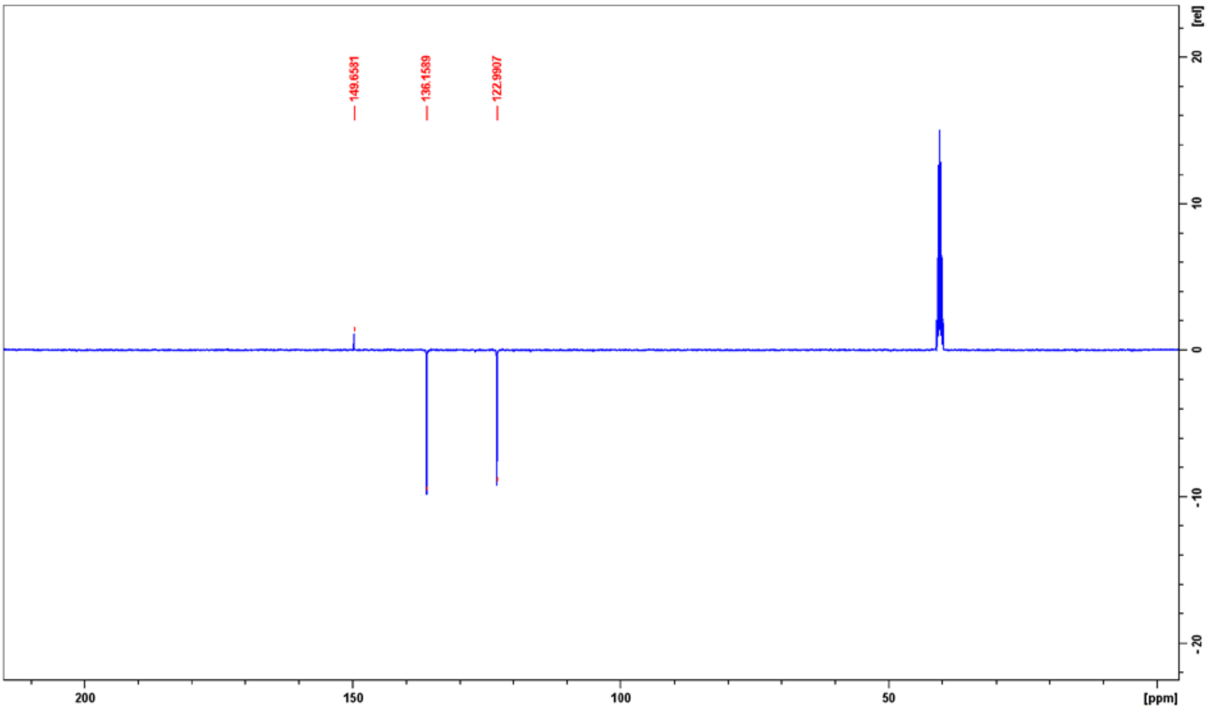

Figure S24. ^{13}C DEPT-Q NMR spectrum of 4-nitrophenyl boronic acid (**3a**) in DMSO-d_6.

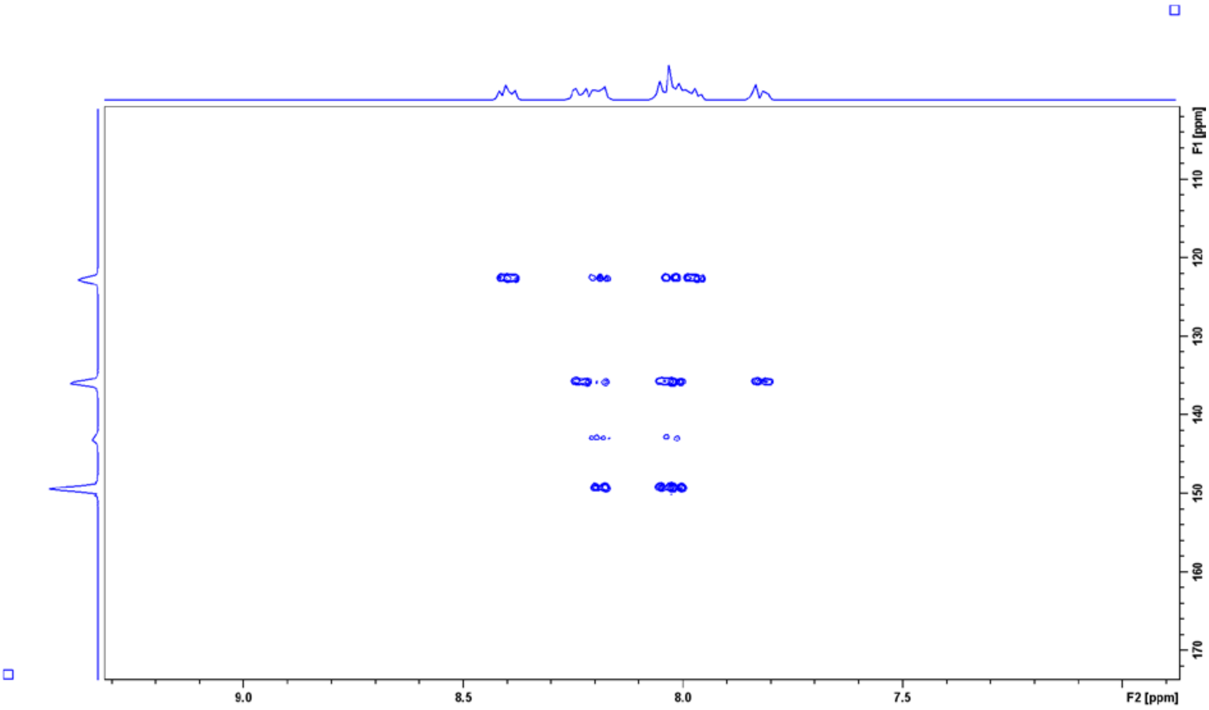

Figure S25. ^{1}H-^{13}C HMBC NMR spectrum of 4-nitrophenyl boronic acid (**3a**) in DMSO-d_6.

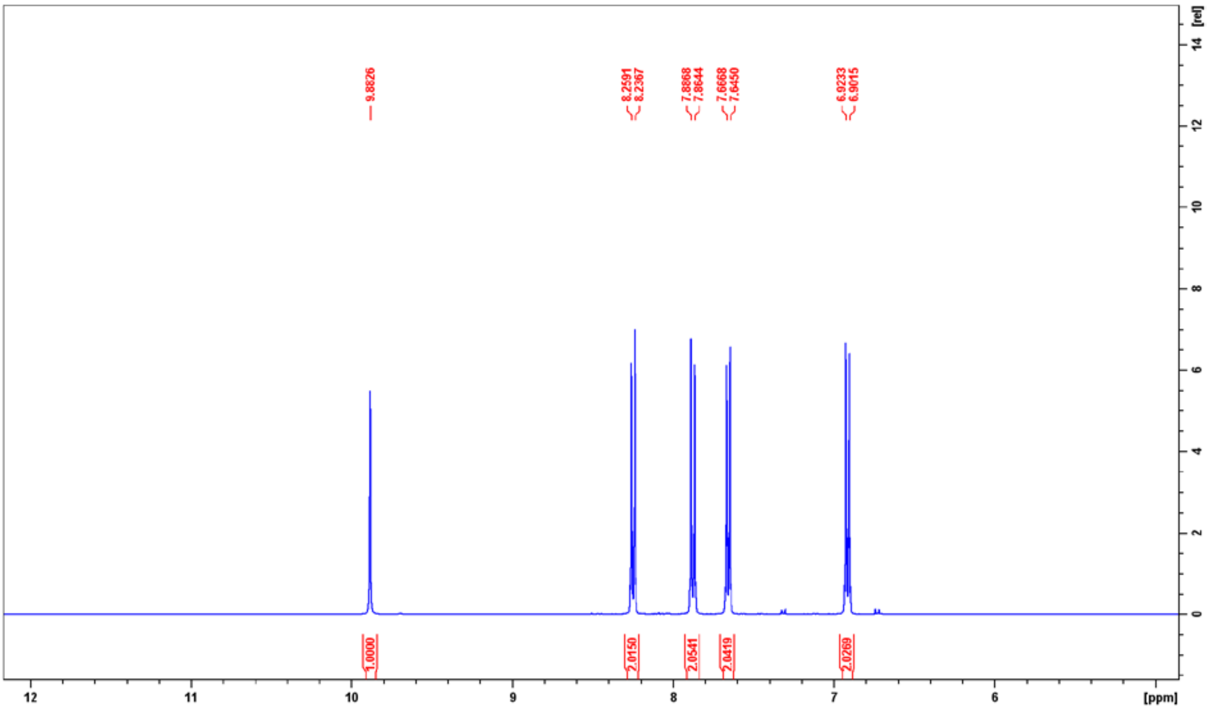

Figure S26. ^{1}H NMR spectrum of 4'-nitrobiphenyl-4-ol (**3b**) in DMSO-d_6.

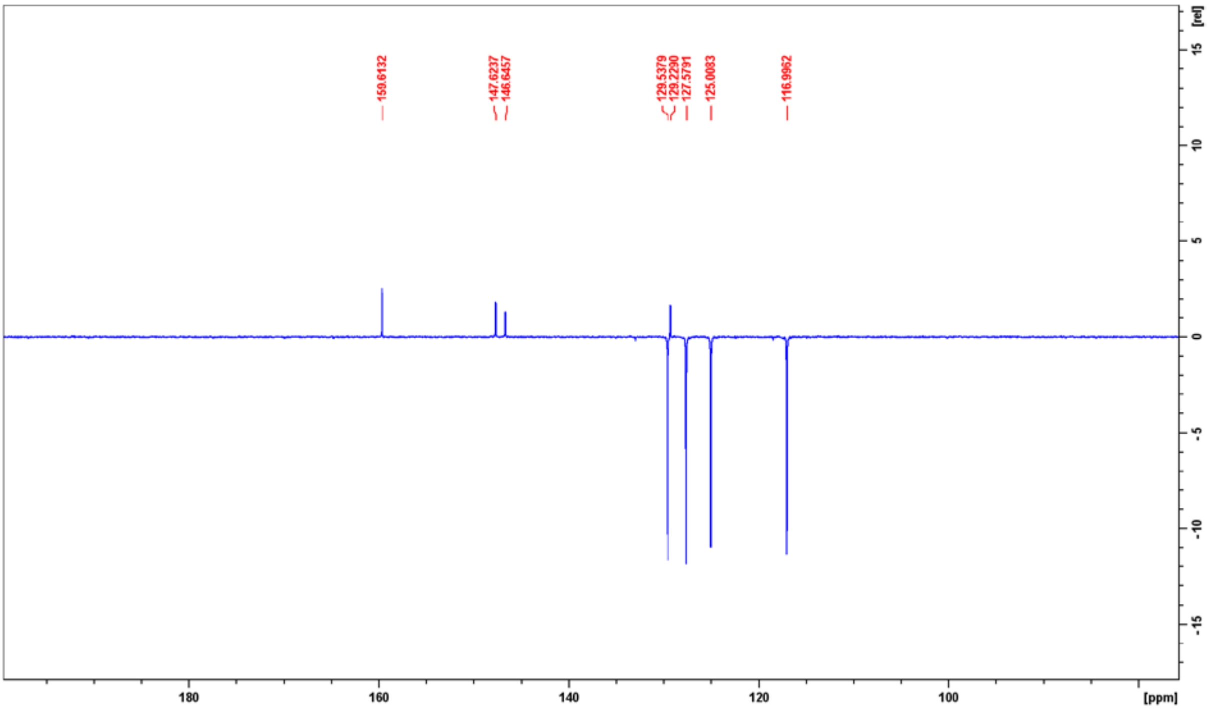

Figure S27. ^{13}C DEPT-Q NMR spectrum of 4'-nitrobiphenyl-4-ol (**3b**) in DMSO-d_6.

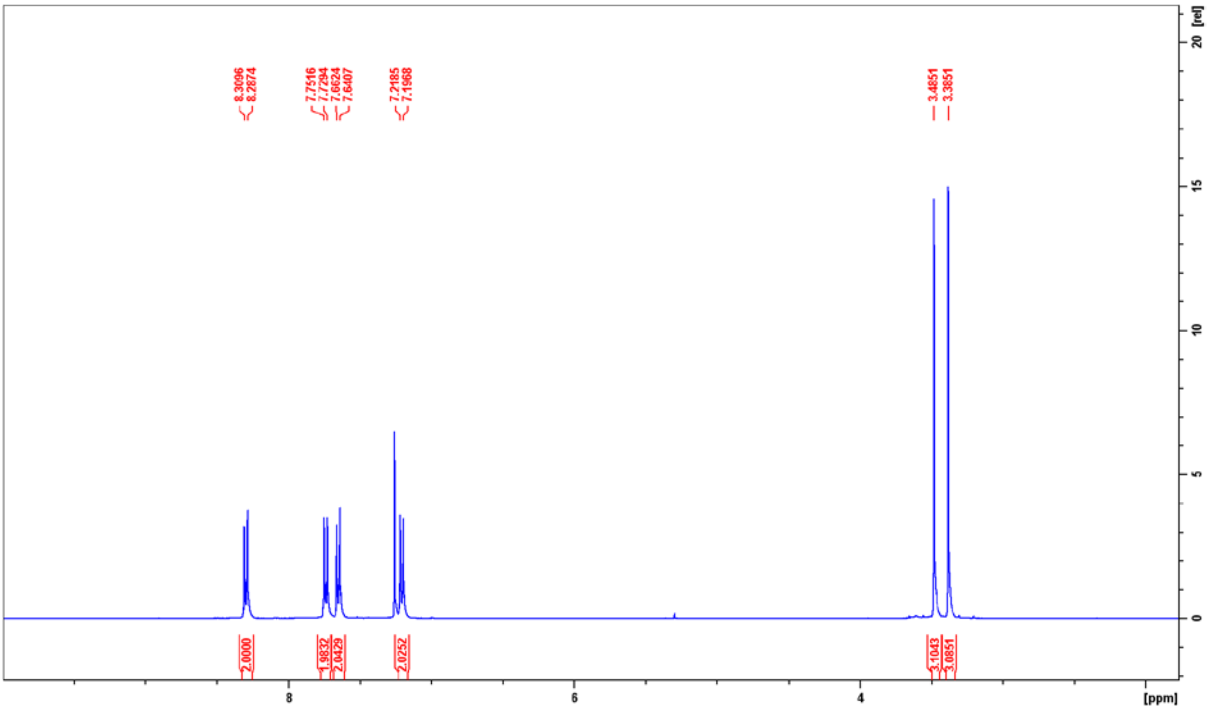

Figure S28. ^1H NMR spectrum of 4'-nitrobiphenyl-4-*O*-(*N*,*N*-dimethylthiocarbamate) (**3c**) in $CDCl_3$.

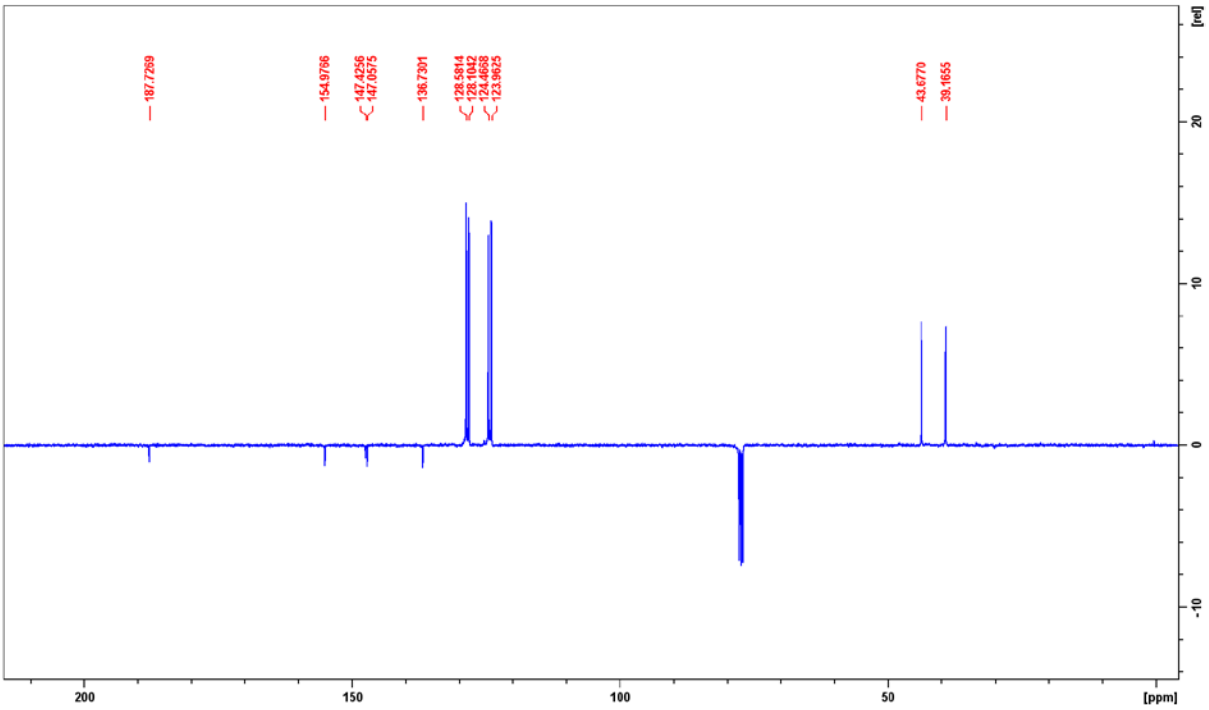

Figure S29. ^{13}C DEPT-Q NMR spectrum of 4'-nitrobiphenyl-4-*O*-(*N*,*N*-dimethylthiocarbamate) (**3c**) in $CDCl_3$.

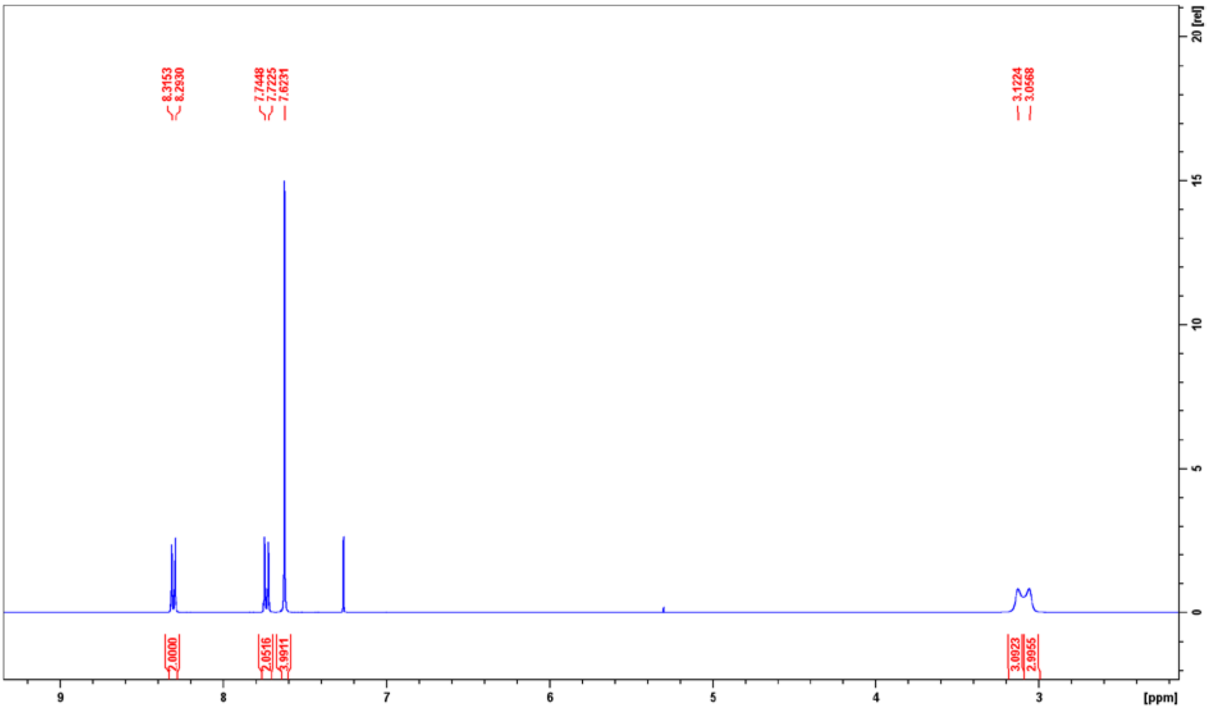

Figure S30. ^{1}H NMR spectrum of 4'-nitrobiphenyl-4-*S*-(*N*,*N*-dimethylthiocarbamate) (**3d**) in $CDCl_3$.

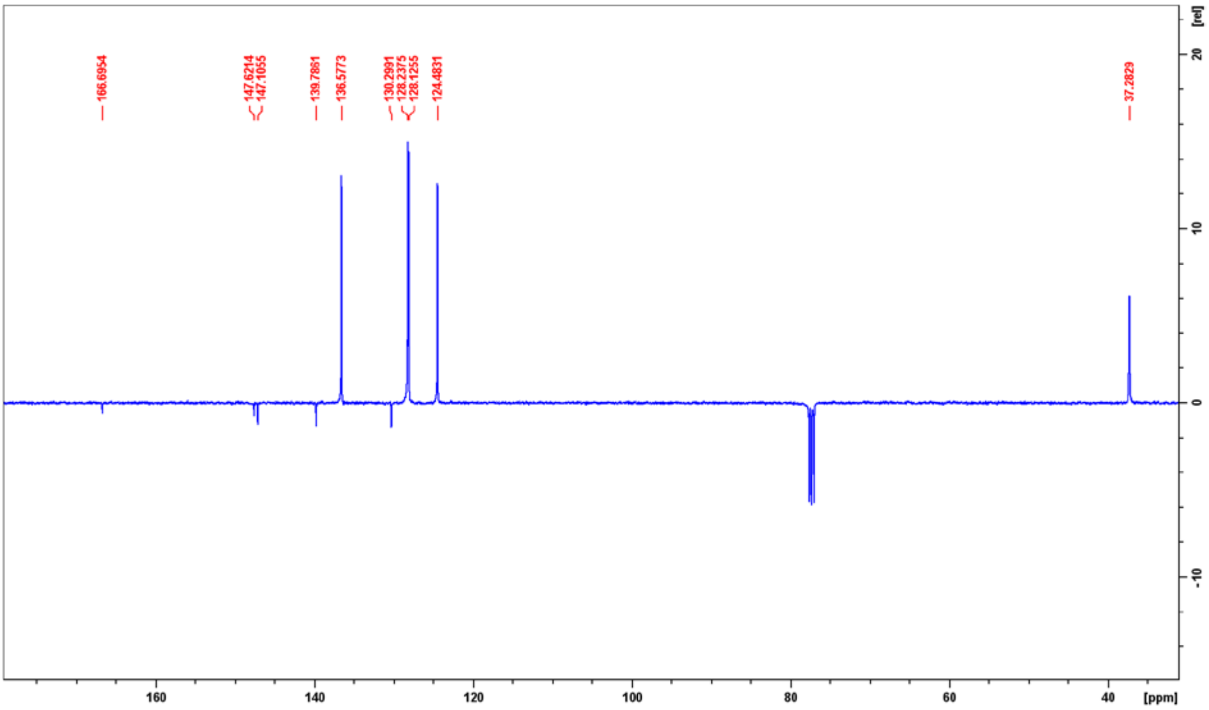

Figure S31. ^{13}C DEPT-Q NMR spectrum of 4'-nitrobiphenyl-4-*S*-(*N*,*N*-dimethylthiocarbamate) (**3d**) in $CDCl_3$.

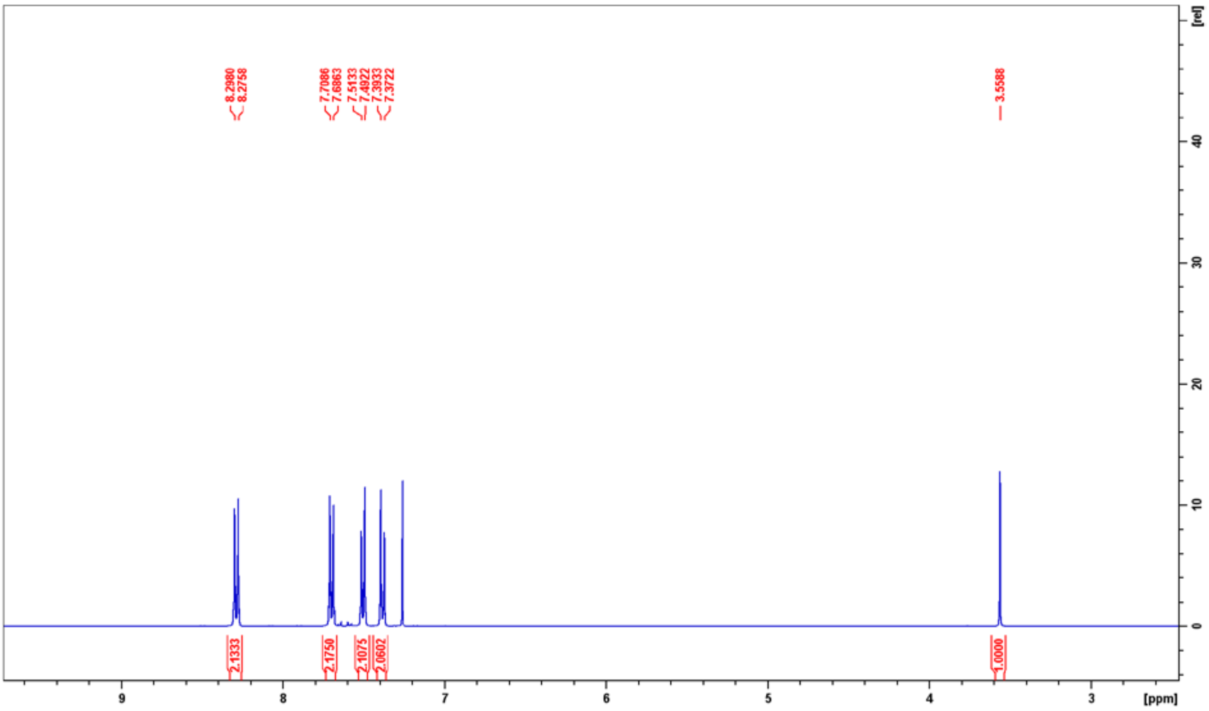

Figure S32. ^{1}H NMR spectrum of 4'-nitrobiphenyl-4-thiol (**3e**) in $CDCl_3$.

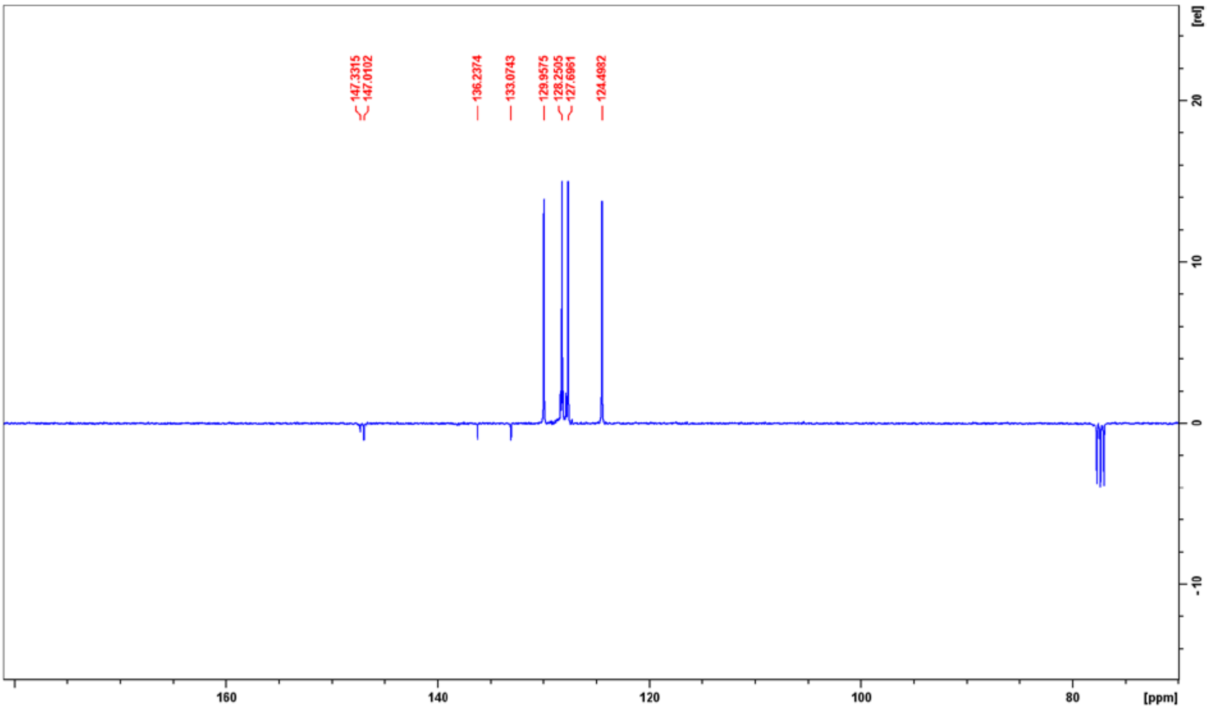

Figure S33. ^{13}C DEPT-Q NMR spectrum of 4'-nitrobiphenyl-4-thiol (**3e**) in $CDCl_3$.

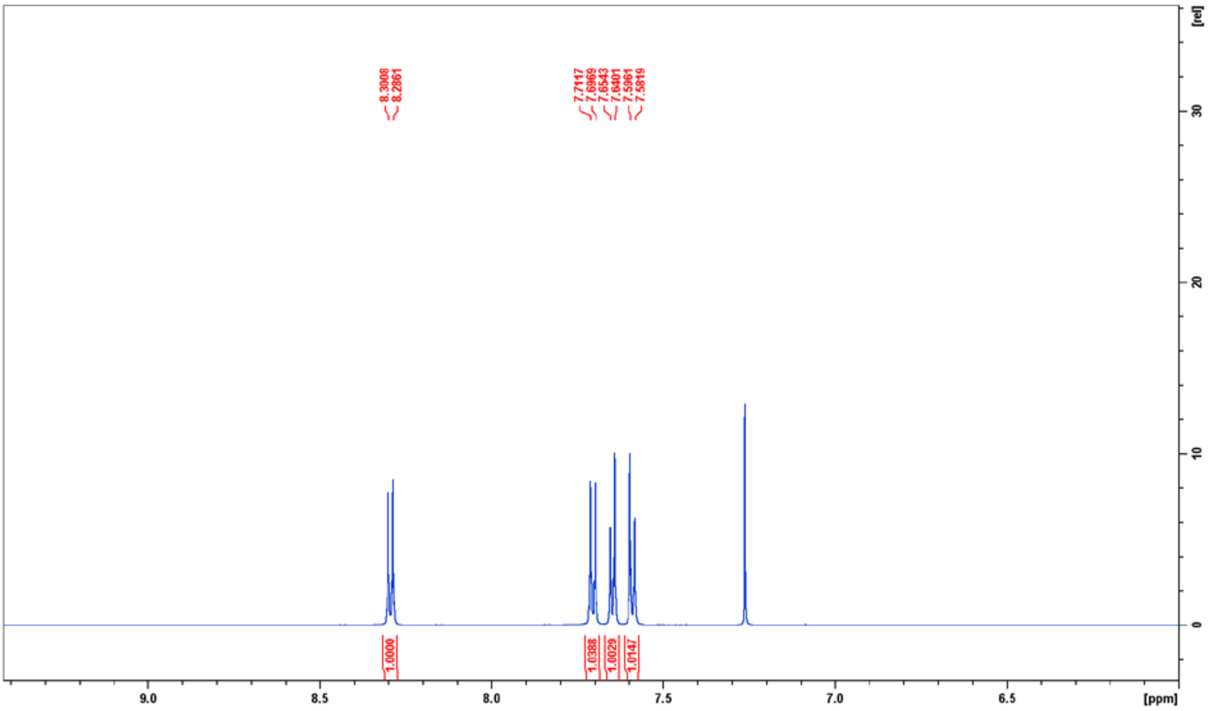

Figure S34. ^{1}H NMR spectrum of 1,2-bis(4'-nitro-[1,1’-biphenyl]-4-yl)disulfane, **NO_2BPDS** (**3f**).

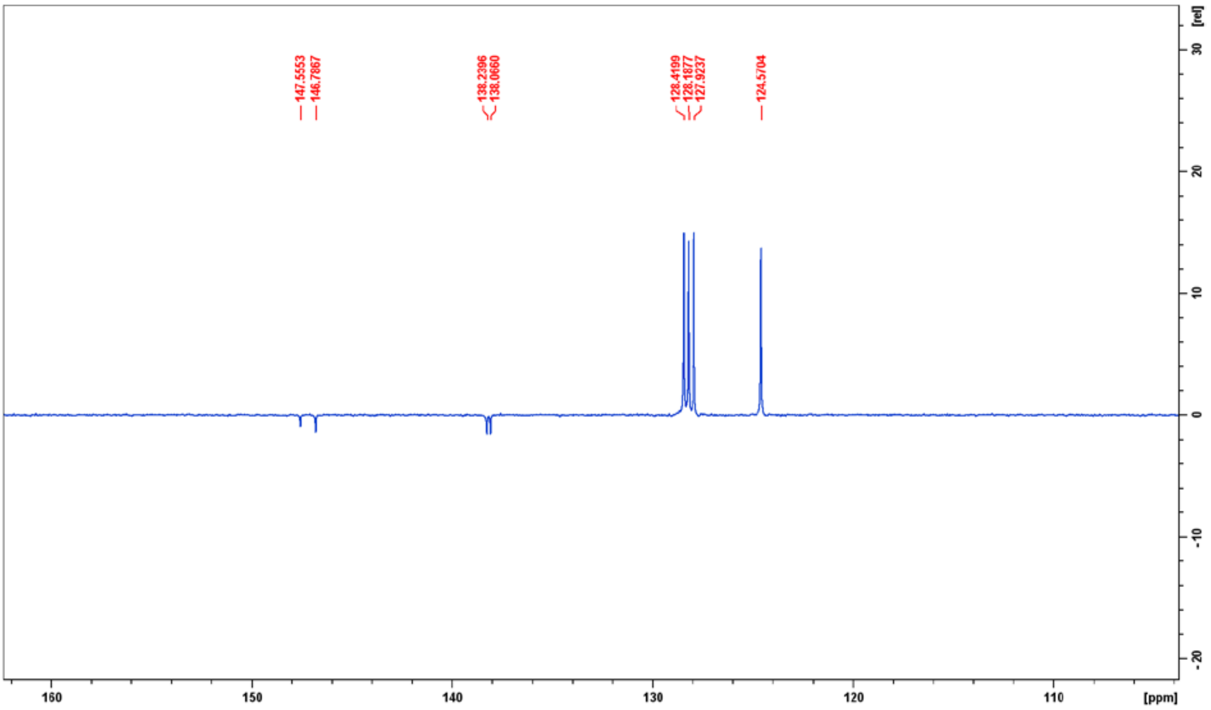

Figure S35. ^{13}C DEPT-Q NMR spectrum of 1,2-bis(4'-nitro-[1,1’-biphenyl]-4-yl)disulfane, **NO2BPDS** (**3f**).

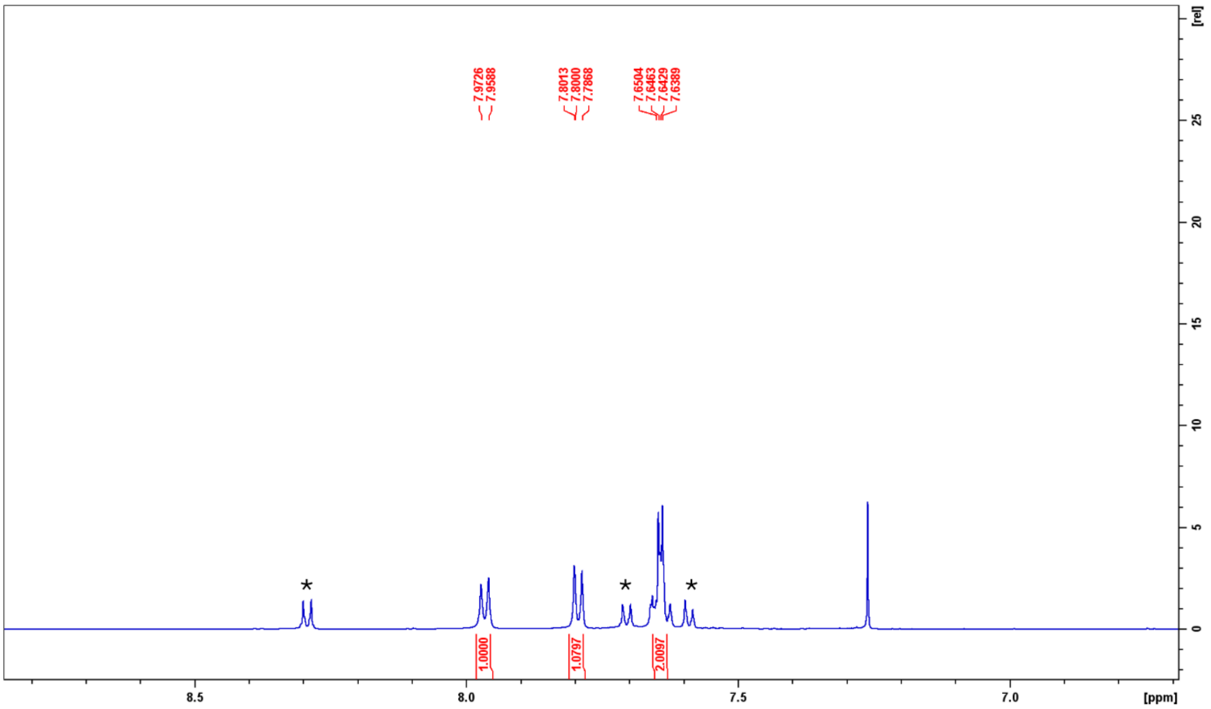

Figure S36. ^{1}H NMR spectrum of 1,2-bis(4'-nitroso-[1,1’-biphenyl]-4-yl)disulfane, **NOBPDS** (**3**) in $CDCl_3$. Signals of impurity (**NO2BPDS** (**3f**)) at 8.29 ppm,7.71 ppm and 7.59 ppm are labeled with *.

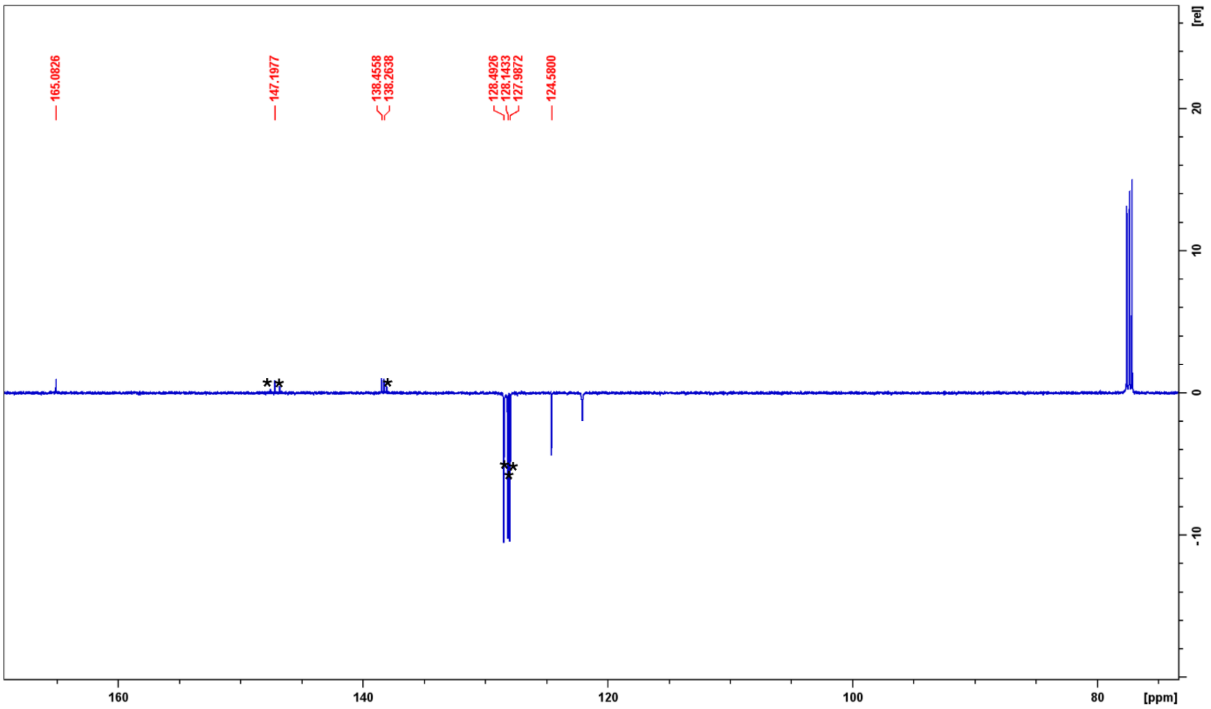

Figure S37. ^{13}C DEPT-Q NMR spectrum of 1,2-bis(4'-nitroso-[1,1'-biphenyl]-4-yl)disulfane, **NOBPDS** (**3**) in $CDCl_3$. Signals of impurity (**NO2BPDS** (**3f**)) at 147.6 ppm, 146.8 ppm, 138.1 ppm, 128.4 ppm, 128.2 ppm and 127.9 ppm are labeled with *.

4. Raman spectra

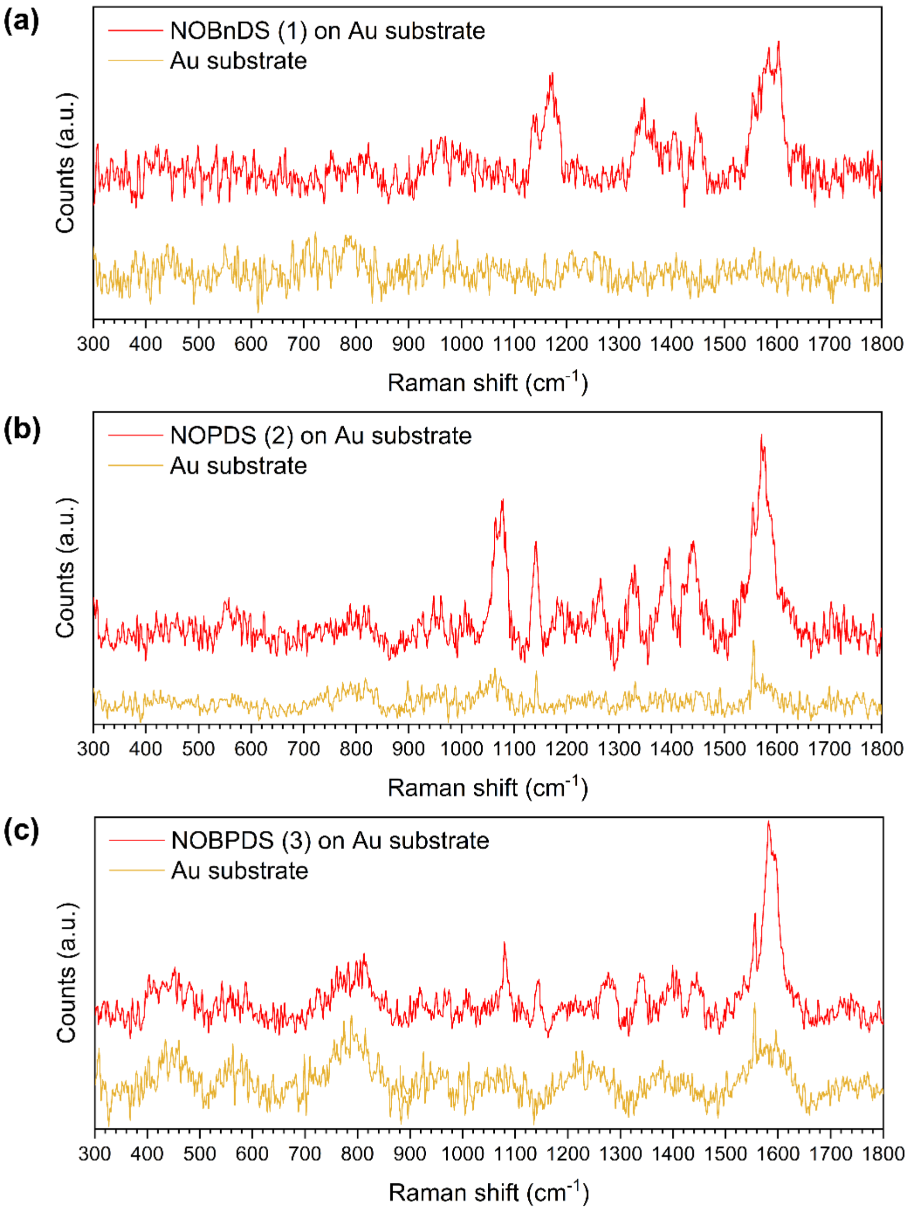

Figure S38. Raman spectra of the (a) **NOBnDS** (**1**), (b) **NOPDS** (**2**) and (c) **NOBPDS** (**3**) films on the Au(111) substrate (in red) compared with the spectra of the bare Au(111) substrate (in yellow).

5. Ellipsometry and contact angle measurements

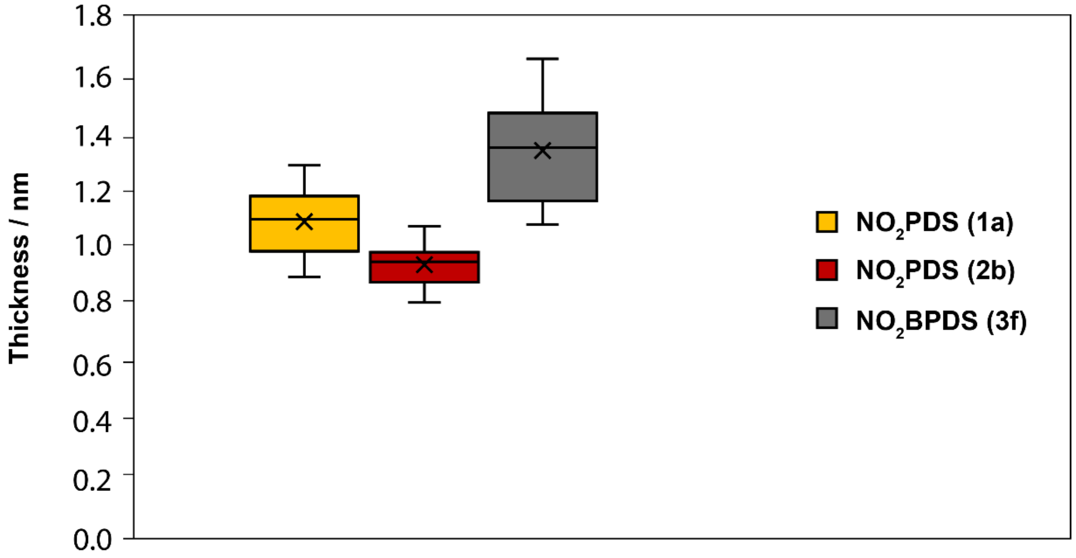

Figure S39. Ellipsometric thicknesses of films produced by immersion of a clean Au(111) substrate in a 1 mM solution of **NO2BnDS** (**1a**), **NO2PDS** (**2b**) and **NO2BPDS** (**3f**) for 24 h. Data are presented as box plots with the boxes displaying 25th to 75th percentiles (the distance between the upper and lower lines of the box is the interquartile range (IQR)), the horizontal lines within the boxes are the median values, the lines outside the boxes represent the range within 1.5 IQR, mean values are depicted by “x” markers.

Table S2. Static water contact angles of **NO2BnDS** (**1a**), **NO2PDS** (**2b**) and **NO2BPDS** (**3f**) films formed on the Au(111) surface after 24 h.

compound	NO_2BnDS (1a)	NO_2PDS (2b)	NO_2BPDS (3f)
average contact angles	50° ± 4°	55° ± 3°	62° ± 2°

6. AFM images

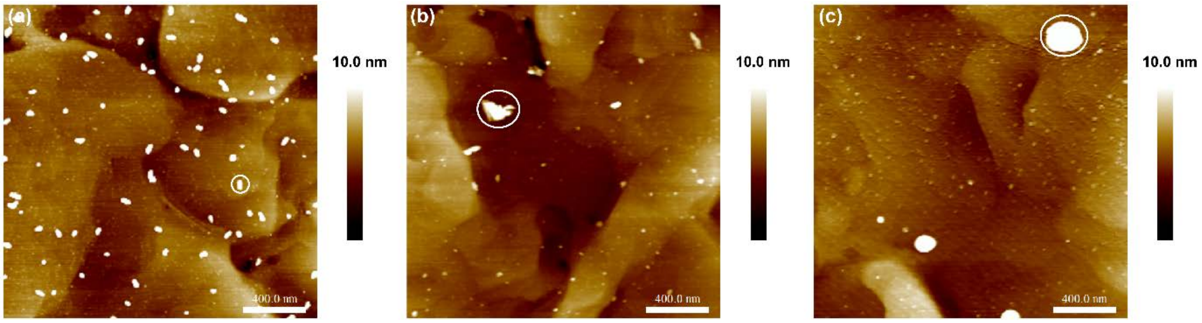

Figure S40. 2 μm × 2 μm AFM images of films prepared by immersion of Au(111) substrate in a 1 mM solution of compound (a) **NO_2BnDS** (**1a**), (b) **NO_2PDS** (**2b**) and (c) **NO_2BPDS** (**3f**) for 24 h. Bright clusters appearing on the surface (marked with white circles) are attributed to environmental contaminants in ambient conditions and surface preparation treatment.

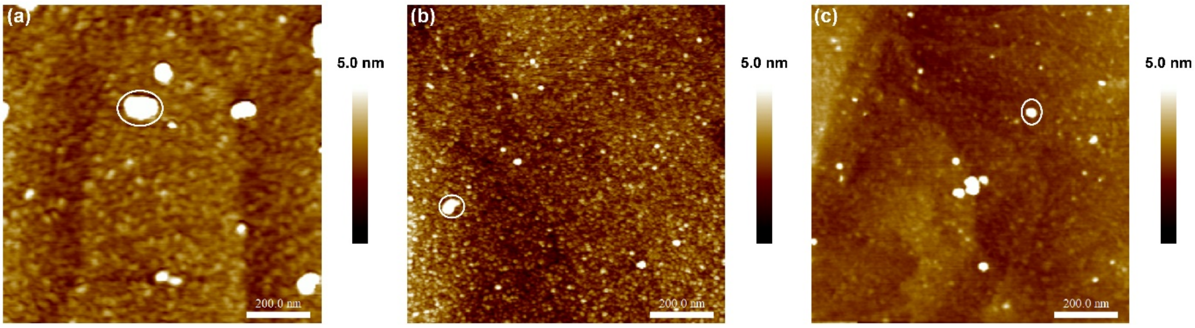

Figure S41. 1 μm × 1 μm AFM images of films prepared by immersion of Au(111) substrate in a 1 mM solution of compound (a) **NOBnDS** (**1**), (b) **NOPDS** (**2**) and (c) **NOBPDS** (**3**) for 48 h. Bright clusters appearing on the surface (marked with white circles) are attributed to environmental contaminants in ambient conditions and surface preparation treatment.

7. STM images

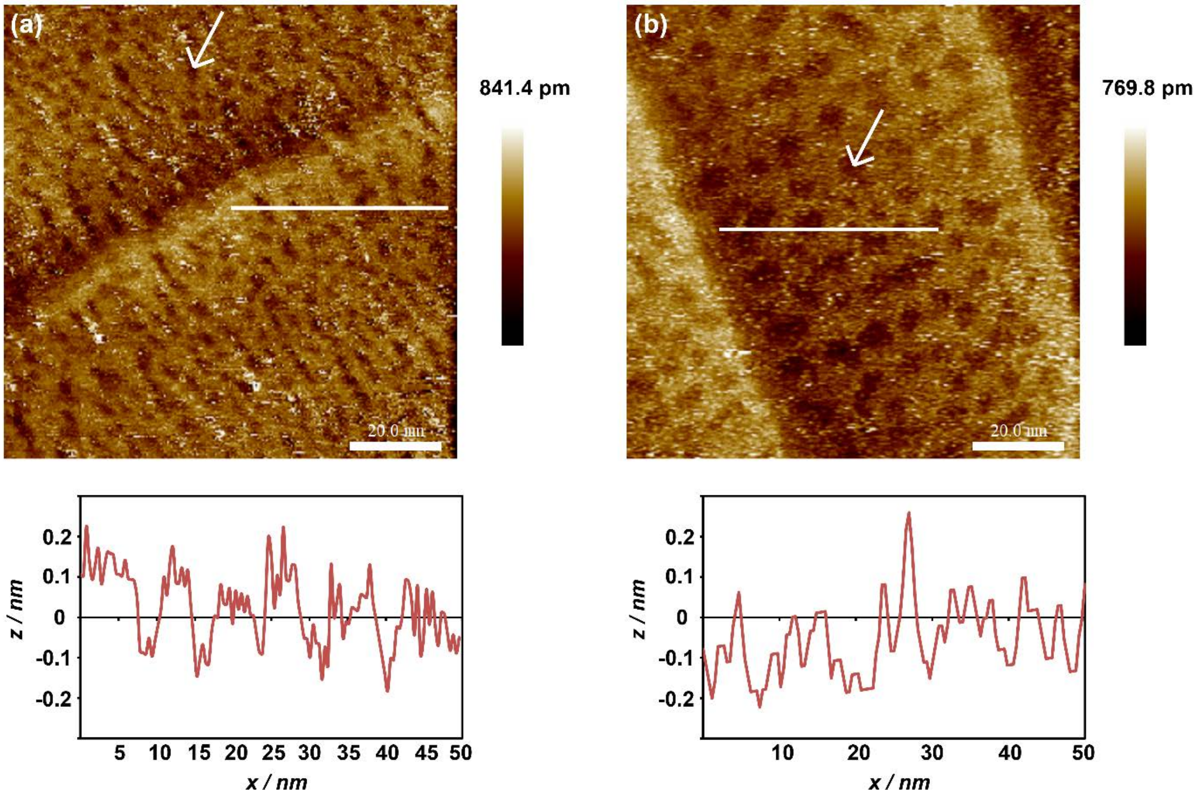

Figure S42. 100 nm × 100 nm STM images of films of compound **NOBnDS** (**1**) prepared by immersion of Au(111) substrate in a 1 mM solution of compound **NOBnDS** (**1**) for 24 h (a) without a subsequent annealing step and (b) followed by post-annealing at 328 K for 2 h. White arrows mark gold vacancies. The height profiles across the selected lines are shown below STM images.

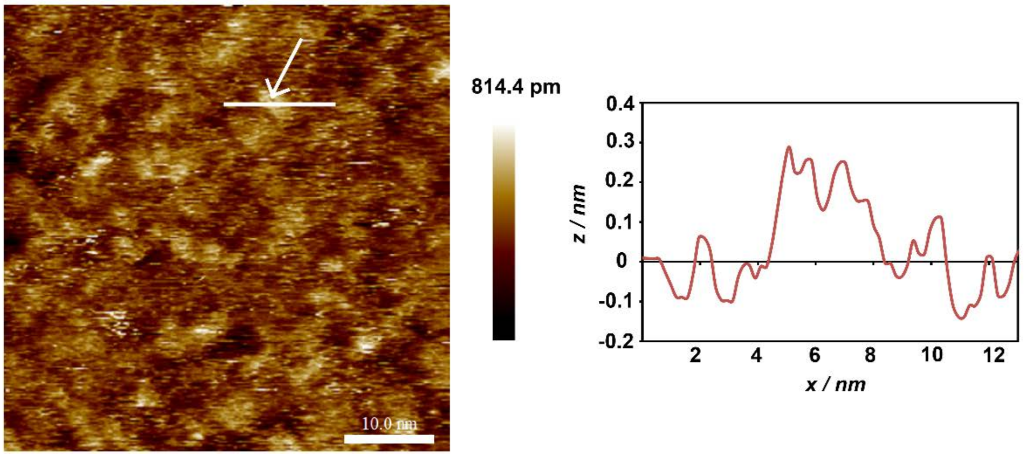

Figure S43. 50 nm × 50 nm STM image of films of compound **NOPDS** (**2**) prepared by immersion of Au(111) substrate in a 1 mM solution for 24 h. White arrow marks gold adatoms. The height profile across the selected line is shown next to the STM image.

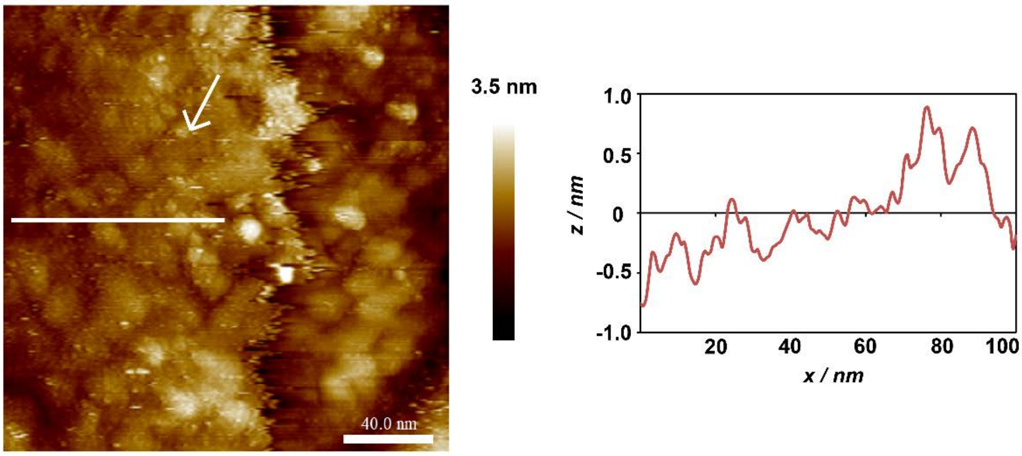

Figure S44. 200 nm × 200 nm STM image of films of compound **NOBPDS** (**3**) prepared by immersion of Au(111) substrate in a 1 mM solution for 24 h. White arrow marks gold adatoms. The height profile across the selected line is shown next to the STM image.

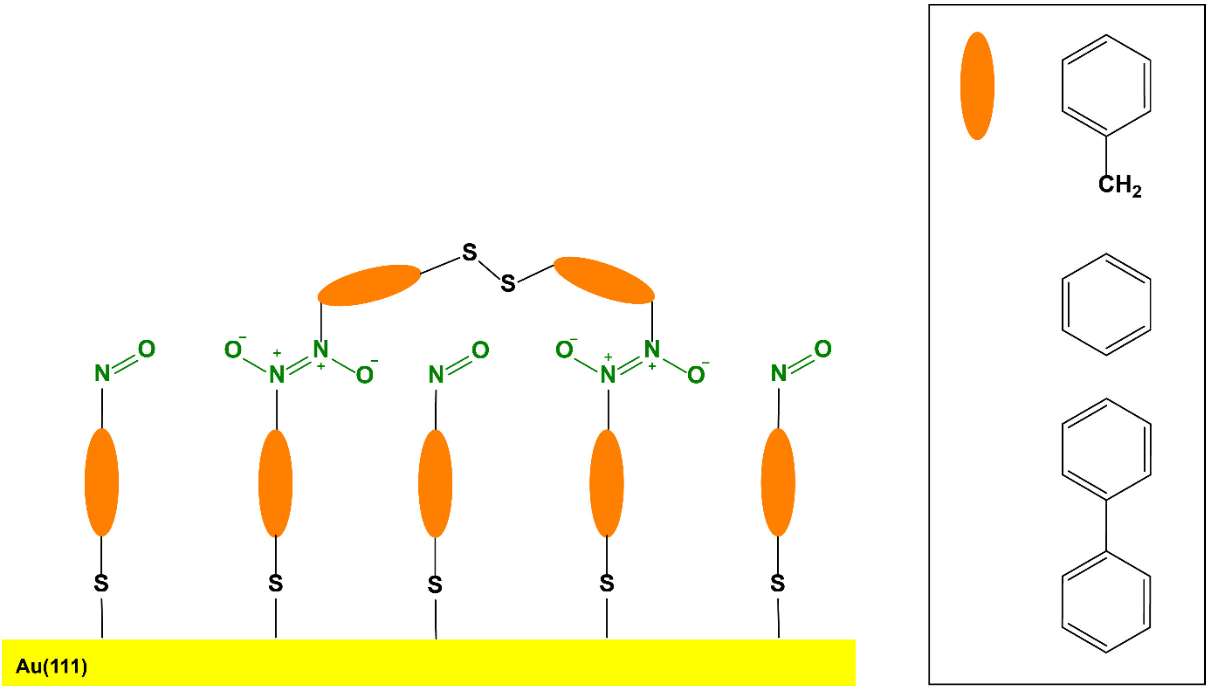

Figure S45. Possible self-polymerization of disulfide-containing nitrosoarenes **NOBnDS** (**1**), **NOPDS** (**2**) and **NOBPDS** (**3**) on the Au(111) surface by creating a bridge through azodioxy bonds on top of the initial SAM. The inset represents different molecular backbones in **NOBnDS** (**1**) (benzyl), **NOPDS** (**2**) (phenyl) and **NOBPDS** (**3**) (biphenyl) depicted by an orange ellipsoid.
